# Impact of the diabetes Canada guideline dissemination strategy on dispensed vascular protective medications for older patients in Ontario, Canada: a linked EMR and administrative data study

**DOI:** 10.1186/s12913-020-05232-3

**Published:** 2020-05-01

**Authors:** Michelle Greiver, Sumeet Kalia, Rahim Moineddin, Simon Chen, Raquel Duchen, Alanna Rigobon

**Affiliations:** 1 Gordon F. Cheesbrough Chair in Family and Community Medicine Research, North York General Hospital, 4001 Leslie Street, LE-140, Toronto, Ontario M2K 1E1 Canada; 2 Department of Family and Community Medicine, Faculty of Medicine, University of Toronto, 500 University Avenue, Toronto, Ontario M5G 1V7 Canada; 3 ICES, G1 06, 2075 Bayview Avenue, Toronto, Ontario M4N 3M5 Canada; 4 Institute of Health Policy, Management and Evaluation, University of Toronto, Toronto, Canada; 5 Faculty of Medicine, University of Toronto, 1 King’s College Cir, Toronto, Ontario M5S 1A8 Canada

**Keywords:** Guideline adherence, Drug utilization, Diabetes mellitus, Primary health care, Electronic health records, Cardiovascular diseases/prevention & control

## Abstract

**Background:**

The 2013 Diabetes Canada guidelines recommended routinely using vascular protective medications for most patients with diabetes. These medications included statins and angiotensin-converting enzyme inhibitors (ACEIs) or angiotensin receptor blockers (ARBs). Antiplatelet agents were only recommended for secondary prevention of cardiovascular disease. Using Electronic Medical Record (EMR) data, we previously found that guideline dissemination efforts were not associated with an increase in the rate of primary care prescriptions of these medications. However, this needs confirmation: patients can receive prescriptions from different sources including specialists and they may not always fill these prescriptions. Using both EMR and administrative health data, we examined whether guideline dissemination impacted the dispensing of vascular protective medications to patients.

**Methods:**

The study population included patients with diabetes aged 66 or over in Ontario, Canada. We created two cohorts using two different approaches: an Electronic Medical Record (EMR) algorithm for diabetes using linked EMR-administrative data and an administrative algorithm using population level administrative data. We examined data from January 2010 to December 2016. Patients with diabetes were deemed to be likely taking a medication (or covered) during a quarter if the daily amount for a dispensed medication would last for at least 75% of days in any given quarter. An interrupted time series analysis was used to assess the proportion of patients covered by each medication class. Proton pump inhibitors (PPIs) were used as a reference.

**Results:**

There was no increase in the rate of change for medication coverage following guideline release in either the EMR or the administrative diabetes cohorts. For statins, the change in trend was − 0.03, *p* = 0.7 (EMR) and − 0.12, *p* = 0.04(administrative). For ACEI/ARBs, this was 0.03, *p* = 0.6 (EMR) and 0, *p* = 1(administrative). For antiplatelets, this was 0.001, *P* = .97 (EMR) and − 0.03, *p* = 0.03 (administrative). The comparator PPI was − 0.07, *p* = 0.4 (EMR) and − 0.11, *p* = 0.002 (administrative).

**Conclusions:**

Using both EMR and administrative health data, we confirmed that the Diabetes Canada 2013 guideline dissemination strategy did not lead to an increased rate of coverage for vascular protective medications. Alternative strategies are needed to effect change in practice.

## Background

Diabetes is a highly prevalent chronic condition, associated with a significantly elevated risk of adverse cardiovascular outcomes, including coronary heart disease and strokes [1]. Effective cardiovascular risk reduction with vascular protective medications for appropriate patients, including statins, angiotensin-converting enzyme inhibitors (ACEIs) or angiotensin receptor blockers (ARBs) and antiplatelet agents is an essential component of diabetes management [2].

To improve the uptake of vascular protective medications, Diabetes Canada issued simplified guidelines in 2013. The 2008 guidelines recommended stratifying patients into risk categories. Patients at high risk should receive one of more of ACEIs or ARBs, statins or possibly ASA [3]. The 2013 guidelines simplified this and no longer require risk stratification. Therefore, the complexity of decisions was reduced as recommendations to start a vascular protective medication were largely based on patient age. The guidelines recommended statins for patients age 40 or over and ACEIs/ARBs for patients age 55 or over. Antiplatelet agents were no longer recommended for the primary prevention of cardiovascular disease but continue to be recommended for secondary prevention [2]. These recommendations were disseminated and implemented nationwide using the Knowledge-to-Action Framework [4]. Interventions including in-person lecture series, conferences, webinars, web-based professional and patient resources such as flow sheets, electronic point of care decision support, a mobile application, and electronic medical record (EMR) templates were rolled out over 24 months starting April 2013 [5]. The recommendations for vascular protection continue to be endorsed through the most recently released 2018 guidelines [6].

We recently examined the uptake of vascular protective medications in Canada following the release of the 2013 Diabetes Canada guidelines, using primary care EMR data from the Canadian Primary Care Sentinel Surveillance Network (CPCSSN), a pan-Canadian EMR-based surveillance system. Primary care providers participating in CPCSSN consent to contribute de-identified EMR data to regional network repositories; patients can opt-out. Data from all participating networks are extracted and processed every 6 months and aggregated in a single central database [7].

We found no improvements in the trend for rates of prescriptions [8]. While the EMR provides information on whether a patient was given a prescription by their primary care provider, it may not provide complete information on whether a patient has filled the prescription or is likely taking the medication (coverage rate). Prescriptions may be received from other providers as patients with diabetes may see both a family physician and an endocrinologist for care. As well, data on the duration of each prescription may be incompletely captured: a medication may be prescribed with several refills that may not be completely captured due to the structure of the EMR. Our previous guideline evaluation used a lag-lead approach to account for such variations in refill protocol and prescription procedures among family physicians [8]. Validating our results using different sources of information under different assumptions is important.

Health administrative databases and clinical data derived from EMRs provide complementary information, and can lead to more complete understanding of patients’ care trajectories [9–11]. Diabetes case definitions based on EMR data have higher sensitivity than those based on administrative data [12, 13]. When validated against chart audits, the sensitivity and specificity for diabetes using CPCSSN data are 95.6 and 97.1% [12]. A widely used, validated Canadian case definition using administrative data has a sensitivity of 86% and a specificity of 97% [14]. EMR data contains information that may not be present in administrative data, including the presence of a diagnosis for the disease in patients’ summary health profiles [13, 15]. Health administrative databases are population based and provide information from sources not captured in EMRs, including hospital visits and specialist encounters [16].

We are now therefore validating our results using data from ICES (formerly the Institute for Clinical Evaluative Sciences), a prescribed entity under Ontario’s privacy legislation authorized to collect and use health care data for the purposes of health system analysis, evaluation and decision support [17]. Our research questions were: did the 2013 Diabetes Canada guidelines influence the coverage of vascular protective medications in patients with diabetes? Was the change in coverage rates obtained using administrative data and linked administrative-EMR data similar to the change in prescription rates obtained using only primary care clinical EMR data? Was a population-based trend in coverage rates similar to our earlier results?

## Methods

This was a retrospective cohort study; we used the Strengthening the Reporting of Observational Studies in Epidemiology guidelines to report the study [18].

### Setting and participants

This study was conducted using data from Ontario, Canada. Ontario’s health care system includes universal, tax-funded coverage for all medically necessary physician services and laboratory tests, with no patient co-payment. About 75% of Ontario’s population is formally registered or enrolled in primary care; each patient is attached to the practice of a family physician. 75% of family physicians practicing comprehensive care use a patient enrollment model. 25% of physicians in patient enrollment models practice in inter-professional Family Health Teams [19].

Two different validated algorithms to define two cohorts of patients living with diabetes were used to examine our data. The first used the CPCSSN (EMR derived) algorithm [20] and the second used the Ontario Diabetes Database (ODD), derived using an administrative data algorithm [14].

### EMR cohort

We used EMR data from the Ontario sites of CPCSSN, extracted up to the end of 2014 and linked at ICES [21]. The four sites included were: Toronto and surroundings, eastern Ontario, Hamilton and London. The CPCSSN algorithm for diabetes [12] was used to identify patients with diabetes in that dataset.

Linked CPCSSN-ICES data from January 1st, 2010 to December 31st 2016 were included. We retrieved data on patients that were present in the CPCSSN dataset, had a valid Ontario health card number, were enrolled or virtually enrolled to a primary care physician and were age 66 or more at the beginning of each quarter of interest. Patients were enrolled in the cohort during or after the quarter containing their first patient-physician encounter as recorded in CPCSSN, if they met the CPCSSN definitions for diabetes at any time prior to each quarter of interest.

### Administrative cohort

We replicated the study using Ontario population based administrative data. Ontario has a population based publicly funded healthcare system; a unique health care number (the Ontario Health Insurance Plan or OHIP) identifies each patient. We used a validated administrative definition for diabetes, the ODD, which is a population-based registry at ICES [14]. The ODD validated algorithm labels cases as diabetes if there are two physician billing claims for diabetes (ICD9 code 250) or one hospitalization discharge record with a diagnostic code of diabetes within 2 years [14]. Patients likely to have gestational diabetes are excluded by not counting billing claims or hospitalizations that appeared 120 days before or 180 days after a hospital record with a diagnosis of pregnancy care or delivery [14].

Similar to the EMR cohort, ICES data from January 1st, 2010 to December 31st 2016 were included. We retrieved data on patients that had a valid Ontario health card number and were age 66 or more at the beginning of each quarter of interest. Patients were enrolled in the cohort if they met the ODD definitions for diabetes at any time prior to each quarter of interest.

Patients were censored from the cohort during the quarter in which they met at least one of the following conditions 1) deceased; 2) last encounter with the healthcare system prior to study end date; 3) loss of eligibility for Ontario’s publicly funded health insurance plan, OHIP; 4) No longer enrolled or virtually enrolled with a primary care physician.

### Variables

The outcome of interest was prescription coverage rates as an indication of whether a patient with diabetes was likely taking an appropriate cardiovascular protective medication in each quarter of interest as per Diabetes Canada guidelines between January 1, 2010 and December 31, 2016. Proton pump inhibitors (PPIs) were used as a comparator for secular trends.

The medications of interest were: 1) Statins; 2) ACE Inhibitors; 3) ARBs; 4) Antiplatelets (only for those with no cardiovascular disease); 5) PPIs.

### Data sources

ICES holds databases on OHIP eligibility, publicly funded medications, and encounters with the health care system such as inpatient and outpatient visits. These datasets were linked using unique encoded identifiers and analyzed at ICES. All patients age 65 or more and covered by OHIP are eligible for publicly funded medication coverage through the Ontario Drug Benefit (ODB) program. The ODB database, available at ICES, contains a copy of information on all ODB eligible medications dispensed in Ontario pharmacies including date dispensed, number of days supplied, cost and Drug Identification Number (DIN), .

Data on medications were obtained using the ODB database. We used the index date corresponding to the date when the medication was dispensed by the pharmacist.

Linked databases at ICES were used to define patient eligibility. These included claims data using the OHIP database for dates of encounters and the Registered Persons Database (RPDB) to define OHIP eligibility, basic demographics and date of death. The Client Agency Program Enrollment (CAPE) tables were used to identify enrollment with a primary care physician; for patients not enrolled, virtual enrollment to a physician was defined through their pattern of care (physician with the majority of primary care billings for a patient). The Ontario Myocardial Infarction Dataset (OMID) [22] were used to define the presence of cardiovascular conditions.

The Johns Hopkins Adjusted Clinical Groups (ACG) method was used to measure disease burden. Adjusted diagnostic groups (ADGs) cluster groups of diagnostic codes (ACGs) to categorize illnesses and predict health care utilization. These diagnostic clusters, or conditions have similar clinical criteria and expected use of resources; there are 32 diagnostic clusters. Each individual patient is assigned ADGs based on their health conditions; a patient can be assigned between zero and 32 ADGs, with more ADGs indicating greater health care needs [23].

ACGs are also used to group patients by expected health care utilization, or Resource Use bands (RUBs). RUBs range from zero (non-users of health care) to five (very high users) [24].

Data from the Ontario portion of CPCSSN have been linked at ICES; linkage rates were 98.7% [21]. The population included in the linked data was slightly older, included a greater proportion of women and was more likely to live in urban areas than the Ontario population. The linked, combined data were used for this study.

### Quantitative variables

An individual was considered to be covered (likely taking a medication) during a given quarter if they were found to have a supply of this medication with a duration overlapping at least 75% of days in that time period. The medication did not have to be dispensed in the given quarter, as the maximum allowed supply in ODB is 100 days. For early refills, we restricted the maximum leftover supply to be 100 days. We grouped ACEis and ARBs in the analysis as they were considered to be equivalent in terms of cardiovascular protection [25]. ARBs are often substituted if ACE is cause side effects such as coughing [25].

This study used an interrupted time series model to determine the impact of the 2013 guidelines on the prescription rates of statin, ACEi/ARB and antiplatelets on a quarterly basis from 2010 to 2016. In particular, the primary outcome included the proportion of eligible patients who had a supply of statin, ACEi/ARB or antiplatelets medication for at least 67 days (75% of days or more) within a 90-day quarter. A total of 13 and 14 quarters were selected before and after the implementation of the Diabetes Canada guidelines in April 2013 (Quarter 2). The intervention effect is assessed using two parameters: (i) a change in trend (i.e. difference in pre-slope and post-slope) and (ii) a change in level (i.e. difference at the beginning of the intervention) [26].

Since the Diabetes Canada guidelines are readily available as a public resource to clinicians, this interrupted time series model was fitted without a roll-in period of the intervention. As part of the sensitivity analyses, the interrupted time series analysis was repeated by excluding patients with no encounters in the EMR (CPCSSN dataset), where there was therefore no opportunity to prescribe medications in primary care.

The analyses were carried out using SAS version 9.4 (SAS Institute. SAS/STAT Software, Version 9.4. SAS, Cary, NC, 2012) and *p*-values < 0.05 were considered to be statistically significant.

## Results

### EMR cohort

The size of the cohort increased from 2010 to 2015 as additional practices were being recruited each year into CPCSSN. The size of the cohort varied from 4385 patients to 7424 patients.

Patient characteristics for the first quarter of each year of interest are provided in Table 1. A table with all quarters is provided as supplementary file 1. Between 94.1 and 97.1% of patients identified using the CPCSSN algorithm were also flagged in the ODD. A greater proportion of patients in the CPCSSN data were in the highest income quintile when compared to those in the administrative data.

**Table 1:** Patient characteristics for the first quarter of each year of interest, EMR cohort

	2010Q1	2011Q1	2012Q1	2013Q1	2014Q1	2015Q1	2016Q1
TOTAL	*N* = 4385	*N* = 5342	*N* = 6071	*N* = 6819	*N* = 7330	*N* = 7424	*N* = 7363
Flagged in ODD	4258 (97.1%)	5167 (96.7%)	5839 (96.2%)	6467 (94.8%)	6914 (94.3%)	6986 (94.1%)	6933 (94.2%)
Have no encounter date*	624 (14.2%)	637 (11.9%)	631 (10.4%)	653 (9.6%)	687 (9.4%)	673 (9.1%)	662 (9.0%)
Age							
Mean ± SD	75.83 ± 6.83	75.78 ± 6.91	75.88 ± 6.97	75.83 ± 7.08	75.77 ± 7.22	75.96 ± 7.29	76.08 ± 7.33
Median (IQR)	75 (70–81)	75 (70–81)	75 (70–81)	75 (70–81)	75 (70–81)	75 (70–81)	75 (70–81)
Sex							
Female	2216 (50.5%)	2694 (50.4%)	3034 (50.0%)	3459 (50.7%)	3733 (50.9%)	3793 (51.1%)	3768 (51.2%)
Male	2169 (49.5%)	2648 (49.6%)	3037 (50.0%)	3360 (49.3%)	3597 (49.1%)	3631 (48.9%)	3595 (48.8%)
Income quintile							
Q1 (lowest)	811 (18.5%)	955 (17.9%)	1072 (17.7%)	1209 (17.7%)	1315 (17.9%)	1331 (17.9%)	1319 (17.9%)
Q2	809 (18.4%)	986 (18.5%)	1131 (18.6%)	1295 (19.0%)	1387 (18.9%)	1401 (18.9%)	1423 (19.3%)
Q3	860 (19.6%)	1064 (19.9%)	1179 (19.4%)	1361 (20.0%)	1453 (19.8%)	1458 (19.6%)	1435 (19.5%)
Q4	934 (21.3%)	1130 (21.2%)	1289 (21.2%)	1410 (20.7%)	1532 (20.9%)	1550 (20.9%)	1531 (20.8%)
Q5 (highest)	970 (22.1%)	1203 (22.5%)	1391 (22.9%)	1531 (22.5%)	1633 (22.3%)	1672 (22.5%)	1642 (22.3%)
Rural	1026 (23.4%)	1270 (23.8%)	1435 (23.6%)	1575 (23.1%)	1680 (22.9%)	1708 (23.0%)	1687 (22.9%)
ADG							
Mean ± SD	7.54 ± 3.86	7.63 ± 3.83	7.70 ± 3.85	7.79 ± 3.87	7.71 ± 3.88	7.68 ± 3.93	7.71 ± 3.92
Median (IQR)	7 (5–10)	7 (5–10)	7 (5–10)	7 (5–10)	7 (5–10)	7 (5–10)	7 (5–10)
RUB							
Mean ± SD	3.65 ± 0.89	3.66 ± 0.90	3.66 ± 0.91	3.68 ± 0.90	3.67 ± 0.90	3.67 ± 0.91	3.66 ± 0.92
Median (IQR)	3 (3–4)	3 (3–4)	3 (3–4)	3 (3–4)	3 (3–4)	3 (3–4)	3 (3–4)
OMID (AMI)	369 (8.4%)	431 (8.1%)	505 (8.3%)	549 (8.1%)	577 (7.9%)	588 (7.9%)	582 (7.9%)
Enrollment status							
Enrolled	4142 (94.5%)	5083 (95.2%)	5774 (95.1%)	6469 (94.9%)	6958 (94.9%)	7046 (94.9%)	6952 (94.4%)
Virtually enrolled	243 (5.5%)	259 (4.8%)	297 (4.9%)	350 (5.1%)	372 (5.1%)	378 (5.1%)	411 (5.6%)
CVD	2856 (65.1%)	3695 (69.2%)	4257 (70.1%)	4901 (71.9%)	5389 (73.5%)	5449 (73.4%)	5386 (73.1%)
CHF	628 (14.3%)	820 (15.4%)	901 (14.8%)	985 (14.4%)	1054 (14.4%)	1091 (14.7%)	1069 (14.5%)
OMID (AMI)	369 (8.4%)	431 (8.1%)	505 (8.3%)	549 (8.1%)	577 (7.9%)	588 (7.9%)	582 (7.9%)
HTN	3610 (82.3%)	4398 (82.3%)	5009 (82.5%)	5635 (82.6%)	6039 (82.4%)	6124 (82.5%)	6089 (82.7%)

ODD: Ontario Diabetes Database; SD: standard deviation; IQR: interquartile range; ADG: adjusted diagnostic groups; RUB: resource utilization band; OMID: Ontario myocardial infarct dataset; AMI: acute myocardial infarct; CVD: cardiovascular disease; CHF: congestive heart failure; HTN: hypertension

*patient had no encounters in the CPCSSN dataset

† RUBs estimate healthcare resource use grouped by morbidity levels: 0 = non user to 5 = very high morbidity

The rates of medications dispensed, where coverage was at least 75% of days within each quarter is shown in Table 2. This table provides the first quarter of each year of interest and the full data are in Supplementary file 2 .

**Table 2:** Rates of medication coverage for patients with diabetes, for the first quarter of each year, EMR cohort

Drug	2010Q1	2011Q1	2012Q1	2013Q1	2014Q1	2015Q1	2016Q1
STATIN	62.87%	64.71%	64.26%	64.82%	63.97%	64.01%	65.12%
ACE INHIBITORS	43.24%	43.02%	41.33%	39.86%	38.72%	37.97%	37.48%
ARB INHIBITORS	21.07%	21.43%	22.25%	22.23%	22.62%	22.43%	22.87%
ACE or ARB	63.40%	63.93%	63.10%	61.80%	61.20%	60.34%	60.36%
ANTIPLATELETS*	9.39%	8.84%	8.48%	7.83%	7.39%	6.58%	6.49%
PPI	21.85%	23.25%	23.79%	24.83%	25.36%	27.32%	27.65%

*patients with a history of myocardial infarct were excluded

ACEi: angiotensin-converting enzyme inhibitor; ARB: angiotensin receptor blockers; PPI: proton pump inhibitor

Medication coverage for patients with at least one encounter in the CPCSSN data is shown in Supplementary file 2.

**Fig. 1:**
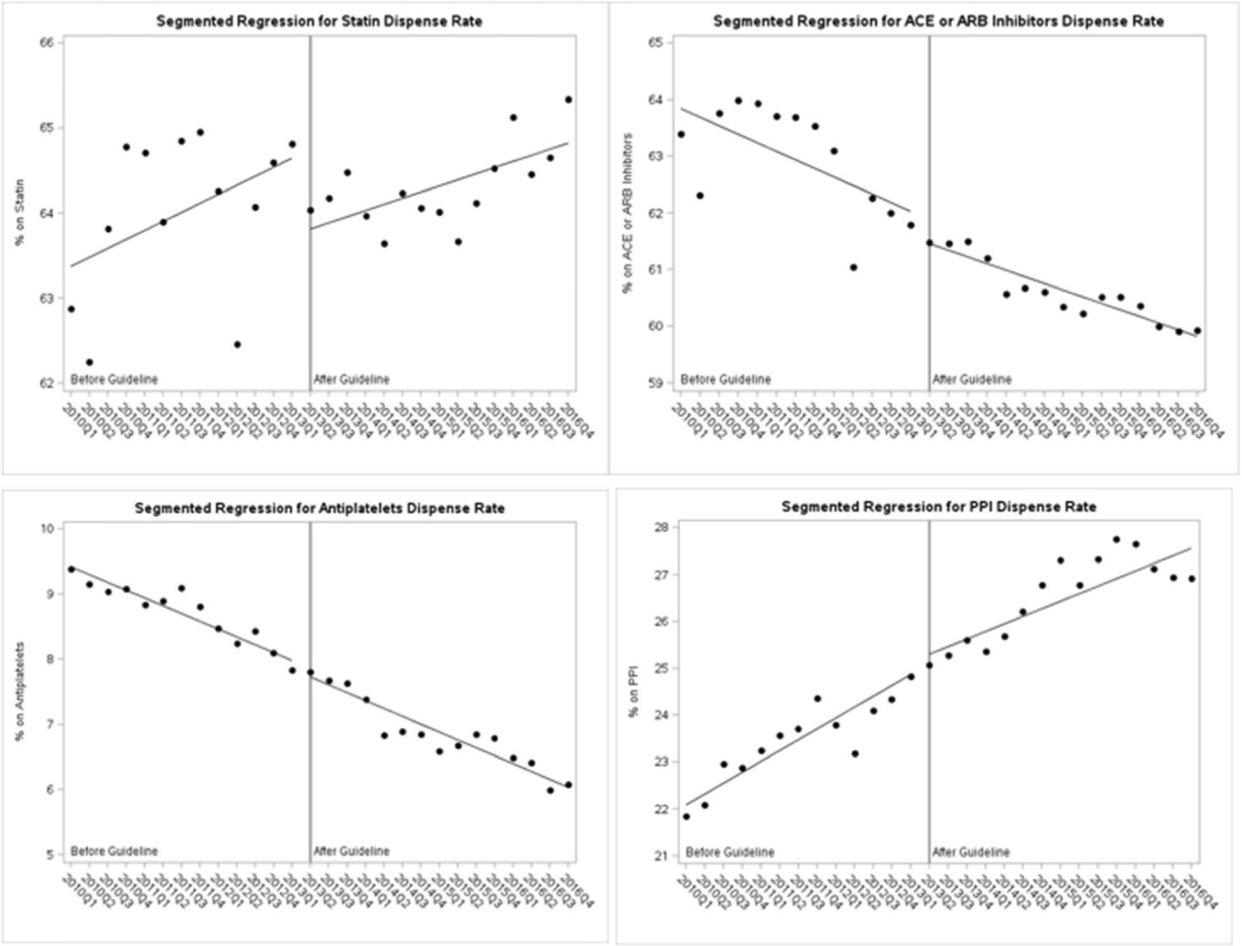
Segmented regression for rate of dispensed medications, EMR data

Results of the interrupted time series analyses are presented in Table 3 and graphically in Fig. 1. There was no significant change in the trend for coverage of any of the vascular protective medications after guideline release. The comparator drug class, PPIs, was increasing prior to guidelines and the rate of increase did not change significantly after.

**Table 3:** Segmented regression analysis for vascular protective medication and comparator coverage 2010 to 2016, EMR cohort

Statin			
Variable	Estimate	SE	***P***value
Baseline prescription rate	63.26	0.52	<.0001
Pre-intervention slope	0.11	0.06	0.11
Change at guideline (2013Q2)	− 0.90	0.61	0.15
Slope change	−0.03	0.09	0.70
ACEi or ARB			
Baseline prescription rate	63.99	0.43	<.0001
Pre-intervention slope	−0.15	0.05	0.01
Change at guideline (2013Q2)	−0.46	0.51	0.38
Slope change	0.03	0.07	0.64
Antiplatelet			
Baseline prescription rate	9.54	0.15	<.0001
Pre-intervention slope	−0.12	0.02	<.0001
Change at guideline (2013Q2)	0.13	0.17	0.5
Slope change	0.001	0.03	0.97
PPI			
Baseline prescription rate	21.84	0.44	<.0001
Pre-intervention slope	0.23	0.05	<.0001
Change at guideline (2013Q2)	0.27	0.43	0.54
Slope change	−0.07	0.08	0.36

*SE* standard error

*ACEi* angiotensin-converting enzyme inhibitor; *ARB* angiotensin receptor blockers; *PPI* proton pump inhibitor

Sensitivity analyses were done, removing all patients with no encounter in the CPCSSN dataset. Results were similar and are provided in Supplementary file 3.

### Administrative cohort

Patient characteristics for the first quarter of each year are provided in Table 4; all quarters are shown in Supplementary file 4. Patient characteristics were similar to those in the EMR cohort; a smaller proportion of patients were enrolled to a family physician.

**Table 4:** Patient characteristics for the first quarter of each year of interest, administrative cohort

	2010Q1	2011Q1	2012Q1	2013Q1	2014Q1	2015Q1	2016Q1
TOTAL	*N* = 443,608	*N* = 466,092	*N* = 488,187	*N* = 513,737	*N* = 544,224	*N* = 571,800	*N* = 599,122
Age							
Mean ± SD	75.60 ± 6.84	75.65 ± 6.91	75.73 ± 6.97	75.70 ± 7.06	75.66 ± 7.15	75.65 ± 7.19	75.67 ± 7.23
Median (IQR)	75 (70–80)	75 (70–80)	75 (70–81)	75 (70–81)	75 (70–81)	75 (70–81)	74 (69–81)
Sex							
Female	223,874 (50.5%)	234,173 (50.2%)	244,748 (50.1%)	256,606 (49.9%)	270,995 (49.8%)	284,190 (49.7%)	297,109 (49.6%)
Male	219,734 (49.5%)	231,919 (49.8%)	243,439 (49.9%)	257,131 (50.1%)	273,229 (50.2%)	287,610 (50.3%)	302,013 (50.4%)
Income quintile							
Q1 (lowest)	95,488 (21.5%)	98,510 (21.1%)	102,097 (20.9%)	106,099 (20.7%)	111,256 (20.4%)	115,641 (20.2%)	120,280 (20.1%)
Q2	97,017 (21.9%)	101,488 (21.8%)	105,691 (21.6%)	110,832 (21.6%)	116,671 (21.4%)	121,898 (21.3%)	127,241 (21.2%)
Q3	88,583 (20.0%)	93,564 (20.1%)	98,297 (20.1%)	103,392 (20.1%)	109,813 (20.2%)	115,603 (20.2%)	121,180 (20.2%)
Q4	85,599 (19.3%)	90,808 (19.5%)	95,852 (19.6%)	101,912 (19.8%)	108,692 (20.0%)	115,226 (20.2%)	121,499 (20.3%)
Q5 (highest)	75,179 (16.9%)	79,898 (17.1%)	84,350 (17.3%)	89,468 (17.4%)	95,666 (17.6%)	101,179 (17.7%)	106,523 (17.8%)
Missing	1742 (0.4%)	1824 (0.4%)	1900 (0.4%)	2034 (0.4%)	2126 (0.4%)	2253 (0.4%)	2399 (0.4%)
Rural	65,408 (14.7%)	68,458 (14.7%)	71,660 (14.7%)	75,092 (14.6%)	79,026 (14.5%)	82,247 (14.4%)	85,540 (14.3%)
ADG							
Mean ± SD	7.90 ± 3.83	7.89 ± 3.83	7.90 ± 3.86	7.89 ± 3.88	7.84 ± 3.92	7.78 ± 3.95	7.81 ± 3.97
Median (IQR)	8 (5–10)	8 (5–10)	8 (5–10)	8 (5–10)	7 (5–10)	7 (5–10)	7 (5–10)
RUB*							
Mean ± SD	3.65 ± 0.90	3.65 ± 0.90	3.65 ± 0.90	3.64 ± 0.90	3.64 ± 0.91	3.64 ± 0.91	3.65 ± 0.92
Median (IQR)	3 (3–4)	3 (3–4)	3 (3–4)	3 (3–4)	3 (3–4)	3 (3–4)	3 (3–4)
OMID (AMI)	37,332 (8.4%)	39,015 (8.4%)	40,729 (8.3%)	42,550 (8.3%)	44,579 (8.2%)	46,463 (8.1%)	48,452 (8.1%)
Enrollment status							
Enrolled	374,436 (84.4%)	399,380 (85.7%)	424,893 (87.0%)	451,284 (87.8%)	482,316 (88.6%)	510,631 (89.3%)	538,094 (89.8%)
Virtually enrolled	69,172 (15.6%)	66,712 (14.3%)	63,294 (13.0%)	62,453 (12.2%)	61,908 (11.4%)	61,169 (10.7%)	61,028 (10.2%)
CVD	381,896 (86.1%)	402,524 (86.4%)	422,519 (86.5%)	444,766 (86.6%)	470,445 (86.4%)	493,342 (86.3%)	515,653 (86.1%)
CHF	70,343 (15.9%)	72,678 (15.6%)	75,397 (15.4%)	78,039 (15.2%)	81,389 (15.0%)	84,822 (14.8%)	87,876 (14.7%)
OMID (AMI)	37,332 (8.4%)	39,015 (8.4%)	40,729 (8.3%)	42,550 (8.3%)	44,579 (8.2%)	46,463 (8.1%)	48,452 (8.1%)
HTN	374,702 (84.5%)	395,440 (84.8%)	415,646 (85.1%)	438,015 (85.3%)	463,672 (85.2%)	486,454 (85.1%)	508,602 (84.9%)

ODD: Ontario Diabetes Database; SD: standard deviation; IQR: interquartile range; ADG: adjusted diagnostic groups; RUB: resource utilization band; OMID: Ontario myocardial infarct dataset; AMI: acute myocardial infarct; CVD: cardiovascular disease; CHF: congestive heart failure; HTN: hypertension

* RUBs estimate healthcare resource use grouped by morbidity levels: 0 = non user to 5 = very high morbidity

The rates of medications dispensed, where coverage was at least 75% of days within each quarter is shown in Table 5. This table provides the first quarter of each year of interest and the full data are in Supplementary file 5. Rates are similar to those in the EMR cohort.

**Table 5:** Rates of medication coverage for patients with diabetes, for the first quarter of each year, 2010 to 2016, administrative cohort

Drug	2010Q1	2011Q1	2012Q1	2013Q1	2014Q1	2015Q1	2016Q1
STATIN	60.91%	62.62%	62.83%	63.76%	63.83%	64.18%	64.48%
ACEi or ARB	61.94%	61.80%	60.87%	60.72%	60.30%	59.72%	59.16%
ANTIPLATELETS*	10.23%	9.97%	9.40%	8.79%	8.14%	7.48%	6.91%
PPI	22.92%	23.91%	24.57%	25.56%	26.14%	26.72%	27.34%

*patients with a history of myocardial infarct were excluded

ACEi: angiotensin-converting enzyme inhibitor; ARB: angiotensin receptor blockers; PPI: proton pump inhibitor

**Fig. 2:**
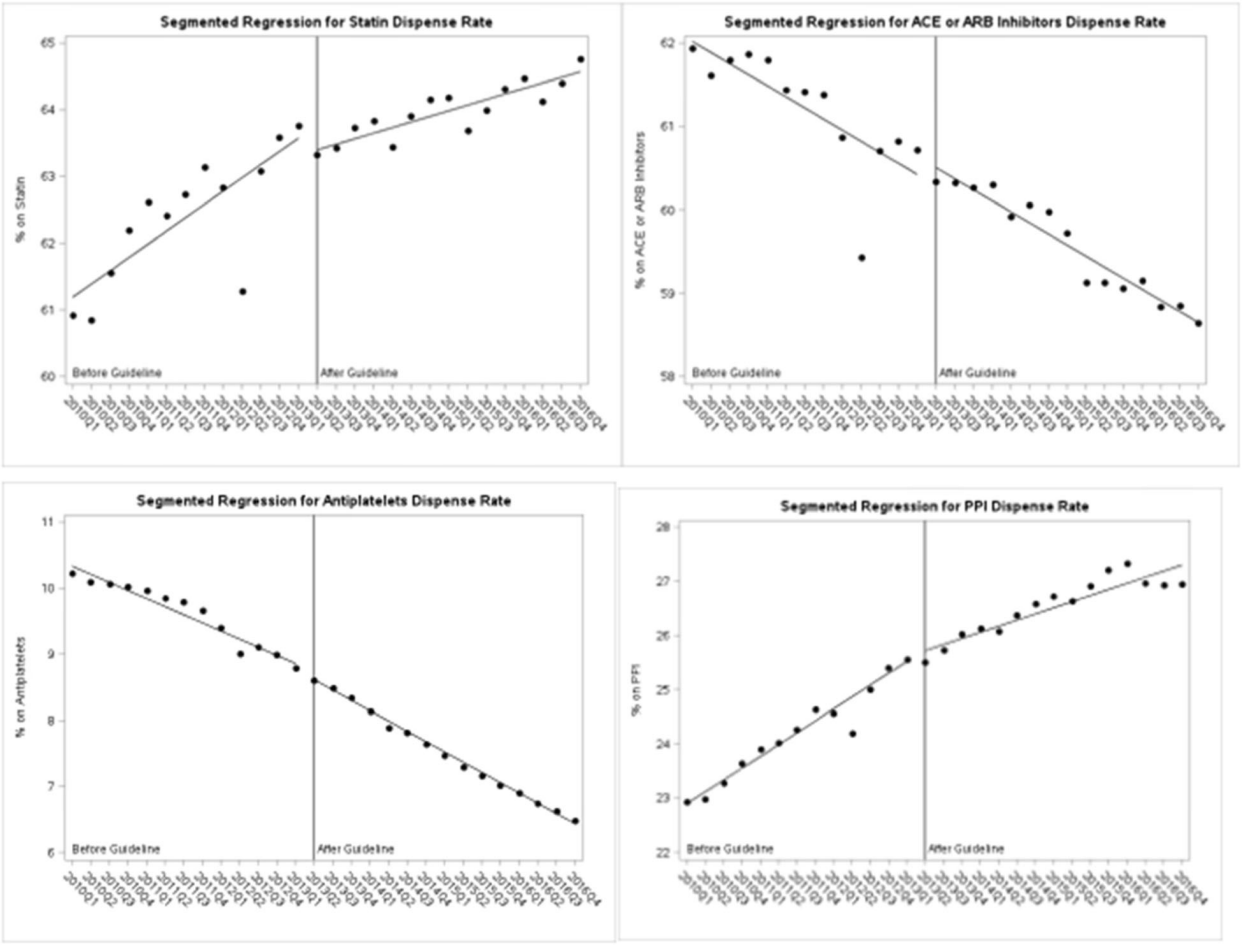
Segmented regression for rate of dispensed medications, administrative data

Results of the interrupted time series analyses are presented in Table 6 and graphically in Fig. 2. Similar to those in the EMR cohort, statin coverage continued to increase while ACEi/ARB coverage decreased. There was no increase in the trend for statins or ACEi/ARBs. The trend in change for antiplatelets and PPIs decreased slightly.

**Table 6:** Segmented regression analysis for vascular protective and comparator medication coverage, 2010 to 2016, administrative cohort

Variable	Estimate	SE	Pvalue
STATIN			
Baseline prescription rate	60.99	0.31	<.0001
Pre-intervention slope	0.20	0.04	<.0001
Change at guideline (2013Q2)	−0.26	0.39	0.51
Slope change	− 0.12	0.05	0.04
ACEI or ARB			
Baseline prescription rate	62.16	0.23	<.0001
Pre-intervention slope	−0.13	0.03	0.0001
Change at guideline (2013Q2)	0.21	0.29	0.46
Slope change	0.00	0.04	0.99
Antiplatelet			
Baseline prescription rate	10.46	0.08	<.0001
Pre-intervention slope	−0.12	0.01	<.0001
Change at guideline (2013Q2)	−0.09	0.08	0.29
Slope change	−0.03	0.01	0.03
PPI			
Baseline prescription rate	22.67	0.18	<.0001
Pre-intervention slope	0.22	0.02	<.0001
Change at guideline (2013Q2)	0.09	0.21	0.67
Slope change	−0.11	0.03	0.002

## Discussion

This study estimated coverage for vascular protective medications in a population of Ontario patients aged 66 or more and living with diabetes. We confirmed using both a primary care EMR cohort and a population-based cohort that the release of guidelines were not associated with an increase in the rate of statin or ACEi/ARB coverage.

Our previous study evaluated the 2013 Diabetes Canada guidelines impact on vascular protective prescriptions using EMR data from CPCSSN, which had limitations in its ability to capture medication coverage, medications prescribed outside of primary care, and duration of prescription given variation in refill protocols [8]. Using administrative data has helped to mitigate these concerns and validate previous findings, which revealed no increase in the rates of statin or ACEi/ARB prescriptions after guideline implementation.

Overall prescription rates from the previous study were lower (52–56% for statins, 42–44% for ACEI/ARBs) compared to the current study (63–65% for statins, 60–64% for ACEI/ARB). Differences may be due to different age criteria (age > 40 years in previous study, age > 65 years for ODB,) or medication coverage. For example, ICES data includes an older cohort (age > 65 years) compared to the previous study, making patients more likely to be prescribed a vascular protective medication given their age and universal prescription coverage. The difference in prescription rates was less marked for antiplatelets, which were prescribed in 10–12% of patients in our previous EMR evaluation compared to 6–10% using administrative dispensing data. We believe this difference may also be related to the increased number of over-the counter antiplatelet medications captured in CPCSSN, which are missing from ODB data.

EMR and health administrative data provide valuable tools for disease monitoring and health surveillance. With the increased use of EMR systems for clinical care, there has been a number of initiatives to link EMR data with administrative data to better identify health conditions, inform health care planning and improve patient outcomes [16, 27–29]. A number of studies demonstrate comparable data from both sources, but note differences in the way data is captured [11] [30]. [31] In terms of prescription data, the EMR contains prescriptions for patients of all ages, as well as those that are prescribed over the counter or those that are not in the provincial drug formulary. Administrative data includes drugs dispensed (rather than prescribed), and from patients who receive provincial drug coverage, often excluding those in younger age categories. Previous research suggests that approximately 14% of medications prescribed do not get filled, leading to a difference in the match rate between prescribing and dispensing data [32]. The combination of both sources thus provides a more robust understanding of medication trends in Canada.

### Limitations

The CPCSSN cohort includes a convenience sample of patients who visit primary care offices, and thus findings may be limited in generalizability. Our data are also derived from the Ontario population, which may not be generalizable to other regions. Patients less than 66 years who are included in the recommendations for vascular protective medications, were not included in the current study as ICES holds medication data from patients who are covered through the ODB program. However, these patients were captured in our previous assessment and demonstrated similar medication trends. This also means that patients without OHIP coverage and those with over the counter prescriptions would not have been captured, however we expect these would represent a small minority of patients overall. Finally, results must be interpreted with caution as administrative data capture medications dispensed, and actual usage of the medications was inferred from these estimates.

## Conclusions

The use of both EMR and administrative data provides a more comprehensive picture of the current state of vascular protection for patients with diabetes in Ontario, Canada. This research helps validate previous findings, which used EMR data and found no improvement in vascular protective prescription rates following the 2013 Diabetes Canada Guidelines implementation and dissemination. Given the similarity in findings between EMR and administrative data, our results suggest that EMR data may offer an additional tool to understand prescription trends across Canada with reasonable accuracy. Future work will be directed at validating the methodology used in analyzing such data, including the lag lead approach from our previous study. Further research efforts are also needed to examine reasons for ongoing vascular protection guideline non-adherence in primary care, including more qualitative assessments.

## Supplementary information

**Additional file 1.** Supplementary file 1. Patient characteristics for all quarters of each year of interest, EMR cohort.

**Additional file 2.** Supplementary file 2: Rates of medication coverage for patients with diabetes, for all quarters of each year, EMR cohort.

**Additional file 3.** Supplementary file 3. Segmented regression analysis for vascular protective medication and comparator coverage, 2010 to 2016, EMR cohort, for patients with at least one encounter.

**Additional file 4.** Supplementary file 4. Patient characteristics for all quarters of interest, administrative cohort.

**Additional file 5.** Supplementary file 5. Rates of medication coverage for patients with diabetes, for all quarters, 2010 to 2016, administrative cohort.

## Data Availability

The dataset from this study is held securely in coded form at the Institute for Clinical Evaluative Sciences (ICES). While data sharing agreements prohibit ICES from making the dataset publicly available, access may be granted to those who meet pre-specified criteria for confidential access, available at https://www.ices.on.ca/DAS. The full dataset creation plan and underlying analytic code are available from the authors upon request, understanding that the programs may rely upon coding templates or macros that are unique to ICES. The corresponding author should be contacted for the dataset creation plan.

## Abbreviations

ACEi: Angiotensin-converting enzyme inhibitor
ARB: Angiotensin receptor blocker
CPCSSN: Canadian Primary Care Sentinel Surveillance Network
EMR: Electronic medical record
ICES: Institute for Clinical Evaluative Sciences
ODD: Ontario Diabetes Database
OHIP: Ontario Health Insurance Plan
PPI: Proton pump inhibitor
