# Supplementary material for: Impact of the diabetes Canada guideline dissemination strategy on dispensed vascular protective medications for older patients in Ontario, Canada: a linked EMR and administrative data study
Source: BMC Health Serv Res. 2020 May 1;20:370. doi: 10.1186/s12913-020-05232-3 (PMC7195730; doi:10.1186/s12913-020-05232-3)
Supplement: Supplementary file 1 — Additional file 1. Supplementary file 1. Patient characteristics for all quarters of each year of interest, EMR cohort. [file 12913_2020_5232_MOESM1_ESM.docx]

Supplementary File 1

Patient characteristics for all quarters of each year of interest, EMR cohort

|  | **2010Q1** | **2010Q2** | **2010Q3** | **2010Q4** | **2011Q1** | **2011Q2** | **2011Q3** | **2011Q4** | **2012Q1** | **2012Q2** | **2012Q3** | **2012Q4** | **2013Q1** | **2013Q2** |
| --- | --- | --- | --- | --- | --- | --- | --- | --- | --- | --- | --- | --- | --- | --- |
| TOTAL | N=4,385 | N=4,561 | N=4,994 | N=5,213 | N=5,342 | N=5,567 | N=5,818 | N=5,952 | N=6,071 | N=6,219 | N=6,477 | N=6,668 | N=6,819 | N=6,970 |
| Flagged in ODD | 4,258 (97.1%) | 4,421 (96.9%) | 4,840 (96.9%) | 5,049 (96.9%) | 5,167 (96.7%) | 5,375 (96.6%) | 5,612 (96.5%) | 5,734 (96.3%) | 5,839 (96.2%) | 5,961 (95.9%) | 6,172 (95.3%) | 6,336 (95.0%) | 6,467 (94.8%) | 6,600 (94.7%) |
| Have no encounter date* | 624 (14.2%) | 631 (13.8%) | 633 (12.7%) | 634 (12.2%) | 637 (11.9%) | 640 (11.5%) | 643 (11.1%) | 635 (10.7%) | 631 (10.4%) | 632 (10.2%) | 644 (9.9%) | 644 (9.7%) | 653 (9.6%) | 666 (9.6%) |
| Age |  |  |  |  |  |  |  |  |  |  |  |  |  |  |
| Mean ± SD | 75.83 ± 6.83 | 75.80 ± 6.84 | 75.75 ± 6.88 | 75.73 ± 6.88 | 75.78 ± 6.91 | 75.82 ± 6.93 | 75.82 ± 6.95 | 75.87 ± 6.94 | 75.88 ± 6.97 | 75.90 ± 7.01 | 75.89 ± 7.02 | 75.88 ± 7.04 | 75.83 ± 7.08 | 75.79 ± 7.12 |
| Median (IQR) | 75 (70-81) | 75 (70-80) | 75 (70-81) | 75 (70-81) | 75 (70-81) | 75 (70-81) | 75 (70-81) | 75 (70-81) | 75 (70-81) | 75 (70-81) | 75 (70-81) | 75 (70-81) | 75 (70-81) | 75 (70-81) |
| Sex |  |  |  |  |  |  |  |  |  |  |  |  |  |  |
| F | 2,216 (50.5%) | 2,286 (50.1%) | 2,527 (50.6%) | 2,636 (50.6%) | 2,694 (50.4%) | 2,810 (50.5%) | 2,917 (50.1%) | 2,973 (49.9%) | 3,034 (50.0%) | 3,126 (50.3%) | 3,262 (50.4%) | 3,372 (50.6%) | 3,459 (50.7%) | 3,526 (50.6%) |
| M | 2,169 (49.5%) | 2,275 (49.9%) | 2,467 (49.4%) | 2,577 (49.4%) | 2,648 (49.6%) | 2,757 (49.5%) | 2,901 (49.9%) | 2,979 (50.1%) | 3,037 (50.0%) | 3,093 (49.7%) | 3,215 (49.6%) | 3,296 (49.4%) | 3,360 (49.3%) | 3,444 (49.4%) |
| Income quintile |  |  |  |  |  |  |  |  |  |  |  |  |  |  |
| Q1 (lowest) | 811 (18.5%) | 837 (18.4%) | 921 (18.4%) | 957 (18.4%) | 955 (17.9%) | 984 (17.7%) | 1,019 (17.5%) | 1,043 (17.5%) | 1,072 (17.7%) | 1,105 (17.8%) | 1,146 (17.7%) | 1,181 (17.7%) | 1,209 (17.7%) | 1,241 (17.8%) |
| Q2 | 809 (18.4%) | 834 (18.3%) | 922 (18.5%) | 965 (18.5%) | 986 (18.5%) | 1,033 (18.6%) | 1,087 (18.7%) | 1,106 (18.6%) | 1,131 (18.6%) | 1,172 (18.8%) | 1,235 (19.1%) | 1,278 (19.2%) | 1,295 (19.0%) | 1,333 (19.1%) |
| Q3 | 860 (19.6%) | 893 (19.6%) | 982 (19.7%) | 1,020 (19.6%) | 1,064 (19.9%) | 1,106 (19.9%) | 1,163 (20.0%) | 1,194 (20.1%) | 1,179 (19.4%) | 1,206 (19.4%) | 1,266 (19.5%) | 1,301 (19.5%) | 1,361 (20.0%) | 1,382 (19.8%) |
| Q4 | 934 (21.3%) | 968 (21.2%) | 1,056 (21.1%) | 1,099 (21.1%) | 1,130 (21.2%) | 1,188 (21.3%) | 1,233 (21.2%) | 1,261 (21.2%) | 1,289 (21.2%) | 1,307 (21.0%) | 1,347 (20.8%) | 1,381 (20.7%) | 1,410 (20.7%) | 1,440 (20.7%) |
| Q5 (highest) | 970 (22.1%) | 1,027 (22.5%) | 1,111 (22.2%) | 1,170 (22.4%) | 1,203 (22.5%) | 1,251 (22.5%) | 1,310 (22.5%) | 1,342 (22.5%) | 1,391 (22.9%) | 1,419 (22.8%) | 1,473 (22.7%) | 1,516 (22.7%) | 1,531 (22.5%) | 1,562 (22.4%) |
| Rural | 1,026 (23.4%) | 1,077 (23.6%) | 1,192 (23.9%) | 1,243 (23.8%) | 1,270 (23.8%) | 1,312 (23.6%) | 1,367 (23.5%) | 1,404 (23.6%) | 1,435 (23.6%) | 1,455 (23.4%) | 1,521 (23.5%) | 1,565 (23.5%) | 1,575 (23.1%) | 1,619 (23.2%) |
| ADG |  |  |  |  |  |  |  |  |  |  |  |  |  |  |
| Mean ± SD | 7.54 ± 3.86 | 7.60 ± 3.86 | 7.62 ± 3.85 | 7.60 ± 3.85 | 7.63 ± 3.83 | 7.66 ± 3.83 | 7.68 ± 3.84 | 7.69 ± 3.86 | 7.70 ± 3.85 | 7.72 ± 3.85 | 7.72 ± 3.88 | 7.74 ± 3.89 | 7.79 ± 3.87 | 7.78 ± 3.88 |
| Median (IQR) | 7 (5-10) | 7 (5-10) | 7 (5-10) | 7 (5-10) | 7 (5-10) | 7 (5-10) | 7 (5-10) | 7 (5-10) | 7 (5-10) | 7 (5-10) | 7 (5-10) | 7 (5-10) | 7 (5-10) | 7 (5-10) |
| RUB |  |  |  |  |  |  |  |  |  |  |  |  |  |  |
| Mean ± SD | 3.65 ± 0.89 | 3.66 ± 0.90 | 3.66 ± 0.91 | 3.66 ± 0.91 | 3.66 ± 0.90 | 3.66 ± 0.91 | 3.67 ± 0.90 | 3.67 ± 0.91 | 3.66 ± 0.91 | 3.67 ± 0.90 | 3.66 ± 0.90 | 3.67 ± 0.91 | 3.68 ± 0.90 | 3.68 ± 0.90 |
| Median (IQR) | 3 (3-4) | 3 (3-4) | 3 (3-4) | 3 (3-4) | 3 (3-4) | 3 (3-4) | 3 (3-4) | 3 (3-4) | 3 (3-4) | 3 (3-4) | 3 (3-4) | 3 (3-4) | 3 (3-4) | 3 (3-4) |
| OMID (AMI) | 369 (8.4%) | 388 (8.5%) | 412 (8.2%) | 424 (8.1%) | 431 (8.1%) | 454 (8.2%) | 474 (8.1%) | 492 (8.3%) | 505 (8.3%) | 513 (8.2%) | 530 (8.2%) | 540 (8.1%) | 549 (8.1%) | 566 (8.1%) |
| Enrollment status |  |  |  |  |  |  |  |  |  |  |  |  |  |  |
| Enrolled | 4,142 (94.5%) | 4,308 (94.5%) | 4,754 (95.2%) | 4,973 (95.4%) | 5,083 (95.2%) | 5,307 (95.3%) | 5,547 (95.3%) | 5,657 (95.0%) | 5,774 (95.1%) | 5,904 (94.9%) | 6,152 (95.0%) | 6,317 (94.7%) | 6,469 (94.9%) | 6,619 (95.0%) |
| Virtually enrolled | 243 (5.5%) | 253 (5.5%) | 240 (4.8%) | 240 (4.6%) | 259 (4.8%) | 260 (4.7%) | 271 (4.7%) | 295 (5.0%) | 297 (4.9%) | 315 (5.1%) | 325 (5.0%) | 351 (5.3%) | 350 (5.1%) | 351 (5.0%) |
|  |  |  |  |  |  |  |  |  |  |  |  |  |  |  |
|  |  |  |  |  |  |  |  |  |  |  |  |  |  |  |
|  | **2010Q1** | **2010Q2** | **2010Q3** | **2010Q4** | **2011Q1** | **2011Q2** | **2011Q3** | **2011Q4** | **2012Q1** | **2012Q2** | **2012Q3** | **2012Q4** | **2013Q1** | **2013Q2** |
| Cardiovascular condition | 2,856 (65.1%) | 3,069 (67.3%) | 3,401 (68.1%) | 3,573 (68.5%) | 3,695 (69.2%) | 3,851 (69.2%) | 3,996 (68.7%) | 4,138 (69.5%) | 4,257 (70.1%) | 4,399 (70.7%) | 4,621 (71.3%) | 4,781 (71.7%) | 4,901 (71.9%) | 5,037 (72.3%) |
| Congestive heart failure | 628 (14.3%) | 659 (14.4%) | 741 (14.8%) | 784 (15.0%) | 820 (15.4%) | 850 (15.3%) | 877 (15.1%) | 890 (15.0%) | 901 (14.8%) | 922 (14.8%) | 964 (14.9%) | 979 (14.7%) | 985 (14.4%) | 1,003 (14.4%) |
| OMID (AMI) | 369 (8.4%) | 388 (8.5%) | 412 (8.2%) | 424 (8.1%) | 431 (8.1%) | 454 (8.2%) | 474 (8.1%) | 492 (8.3%) | 505 (8.3%) | 513 (8.2%) | 530 (8.2%) | 540 (8.1%) | 549 (8.1%) | 566 (8.1%) |
| Hypertension | 3,610 (82.3%) | 3,740 (82.0%) | 4,104 (82.2%) | 4,282 (82.1%) | 4,398 (82.3%) | 4,582 (82.3%) | 4,782 (82.2%) | 4,900 (82.3%) | 5,009 (82.5%) | 5,123 (82.4%) | 5,338 (82.4%) | 5,511 (82.6%) | 5,635 (82.6%) | 5,754 (82.6%) |

ODD: Ontario Diabetes Database; SD: standard deviation; IQR: interquartile range; ADG: adjusted diagnostic groups; RUB: resource utilization band; OMID: Ontario myocardial infarct dataset; AMI: acute myocardial infarct

*patient had no encounters in the CPCSSN dataset

† RUBs estimate healthcare resource use grouped by morbidity levels: 0=non user to 5 = very high morbidity

|  | **2013Q3** | **2013Q4** | **2014Q1** | **2014Q2** | **2014Q3** | **2014Q4** | **2015Q1** | **2015Q2** | **2015Q3** | **2015Q4** | **2016Q1** | **2016Q2** | **2016Q3** | **2016Q4** |
| --- | --- | --- | --- | --- | --- | --- | --- | --- | --- | --- | --- | --- | --- | --- |
| TOTAL | N=7,128 | N=7,249 | N=7,330 | N=7,431 | N=7,449 | N=7,456 | N=7,424 | N=7,418 | N=7,410 | N=7,385 | N=7,363 | N=7,378 | N=7,377 | N=7,359 |
| Flagged in ODD | 6,742 (94.6%) | 6,851 (94.5%) | 6,914 (94.3%) | 6,990 (94.1%) | 7,006 (94.1%) | 7,014 (94.1%) | 6,986 (94.1%) | 6,980 (94.1%) | 6,975 (94.1%) | 6,950 (94.1%) | 6,933 (94.2%) | 6,948 (94.2%) | 6,941 (94.1%) | 6,927 (94.1%) |
| Have no encounter date* | 674 (9.5%) | 684 (9.4%) | 687 (9.4%) | 689 (9.3%) | 686 (9.2%) | 682 (9.1%) | 673 (9.1%) | 673 (9.1%) | 672 (9.1%) | 666 (9.0%) | 662 (9.0%) | 671 (9.1%) | 664 (9.0%) | 661 (9.0%) |
| Age |  |  |  |  |  |  |  |  |  |  |  |  |  |  |
| Mean ± SD | 75.76 ± 7.15 | 75.76 ± 7.18 | 75.77 ± 7.22 | 75.80 ± 7.23 | 75.88 ± 7.26 | 75.94 ± 7.28 | 75.96 ± 7.29 | 75.99 ± 7.31 | 76.01 ± 7.31 | 76.08 ± 7.33 | 76.08 ± 7.33 | 76.09 ± 7.35 | 76.12 ± 7.36 | 76.16 ± 7.38 |
| Median (IQR) | 75 (70-81) | 75 (70-81) | 75 (70-81) | 75 (70-81) | 75 (70-81) | 75 (70-81) | 75 (70-81) | 75 (70-81) | 75 (70-81) | 75 (70-81) | 75 (70-81) | 75 (70-81) | 75 (70-82) | 75 (70-82) |
| Sex |  |  |  |  |  |  |  |  |  |  |  |  |  |  |
| Female | 3,618 (50.8%) | 3,681 (50.8%) | 3,733 (50.9%) | 3,779 (50.9%) | 3,798 (51.0%) | 3,803 (51.0%) | 3,793 (51.1%) | 3,786 (51.0%) | 3,786 (51.1%) | 3,784 (51.2%) | 3,768 (51.2%) | 3,776 (51.2%) | 3,779 (51.2%) | 3,761 (51.1%) |
| Male | 3,510 (49.2%) | 3,568 (49.2%) | 3,597 (49.1%) | 3,652 (49.1%) | 3,651 (49.0%) | 3,653 (49.0%) | 3,631 (48.9%) | 3,632 (49.0%) | 3,624 (48.9%) | 3,601 (48.8%) | 3,595 (48.8%) | 3,602 (48.8%) | 3,598 (48.8%) | 3,598 (48.9%) |
| Income quintile |  |  |  |  |  |  |  |  |  |  |  |  |  |  |
| Q1 (lowest) | 1,265 (17.7%) | 1,288 (17.8%) | 1,315 (17.9%) | 1,321 (17.8%) | 1,324 (17.8%) | 1,325 (17.8%) | 1,331 (17.9%) | 1,316 (17.7%) | 1,311 (17.7%) | 1,306 (17.7%) | 1,319 (17.9%) | 1,328 (18.0%) | 1,325 (18.0%) | 1,321 (18.0%) |
| Q2 | 1,360 (19.1%) | 1,385 (19.1%) | 1,387 (18.9%) | 1,410 (19.0%) | 1,408 (18.9%) | 1,411 (18.9%) | 1,401 (18.9%) | 1,415 (19.1%) | 1,413 (19.1%) | 1,410 (19.1%) | 1,423 (19.3%) | 1,422 (19.3%) | 1,424 (19.3%) | 1,424 (19.4%) |
| Q3 | 1,421 (19.9%) | 1,442 (19.9%) | 1,453 (19.8%) | 1,470 (19.8%) | 1,472 (19.8%) | 1,469 (19.7%) | 1,458 (19.6%) | 1,455 (19.6%) | 1,462 (19.7%) | 1,456 (19.7%) | 1,435 (19.5%) | 1,430 (19.4%) | 1,429 (19.4%) | 1,421 (19.3%) |
| Q4 | 1,480 (20.8%) | 1,509 (20.8%) | 1,532 (20.9%) | 1,559 (21.0%) | 1,564 (21.0%) | 1,567 (21.0%) | 1,550 (20.9%) | 1,547 (20.9%) | 1,548 (20.9%) | 1,542 (20.9%) | 1,531 (20.8%) | 1,541 (20.9%) | 1,546 (21.0%) | 1,541 (20.9%) |
| Q5 (highest) | 1,588 (22.3%) | 1,610 (22.2%) | 1,633 (22.3%) | 1,661 (22.4%) | 1,671 (22.4%) | 1,674 (22.5%) | 1,672 (22.5%) | 1,673 (22.6%) | 1,663 (22.4%) | 1,658 (22.5%) | 1,642 (22.3%) | 1,645 (22.3%) | 1,641 (22.2%) | 1,640 (22.3%) |
| Missing | 14 (0.2%) | 15 (0.2%) | 10 (0.1%) | 10 (0.1%) | 10 (0.1%) | 10 (0.1%) | 12 (0.2%) | 12 (0.2%) | 13 (0.2%) | 13 (0.2%) | 13 (0.2%) | 12 (0.2%) | 12 (0.2%) | 12 (0.2%) |
| Rural | 1,673 (23.5%) | 1,700 (23.5%) | 1,680 (22.9%) | 1,706 (23.0%) | 1,714 (23.0%) | 1,714 (23.0%) | 1,708 (23.0%) | 1,713 (23.1%) | 1,720 (23.2%) | 1,720 (23.3%) | 1,687 (22.9%) | 1,701 (23.1%) | 1,712 (23.2%) | 1,712 (23.3%) |
| ADG |  |  |  |  |  |  |  |  |  |  |  |  |  |  |
| Mean ± SD | 7.75 ± 3.87 | 7.74 ± 3.87 | 7.71 ± 3.88 | 7.68 ± 3.87 | 7.71 ± 3.91 | 7.73 ± 3.92 | 7.68 ± 3.93 | 7.70 ± 3.93 | 7.70 ± 3.91 | 7.72 ± 3.92 | 7.71 ± 3.92 | 7.75 ± 3.91 | 7.74 ± 3.92 | 7.74 ± 3.94 |
| Median (IQR) | 7 (5-10) | 7 (5-10) | 7 (5-10) | 7 (5-10) | 7 (5-10) | 7 (5-10) | 7 (5-10) | 7 (5-10) | 7 (5-10) | 7 (5-10) | 7 (5-10) | 7 (5-10) | 7 (5-10) | 7 (5-10) |
| RUB |  |  |  |  |  |  |  |  |  |  |  |  |  |  |
| Mean ± SD | 3.67 ± 0.90 | 3.67 ± 0.90 | 3.67 ± 0.90 | 3.67 ± 0.90 | 3.68 ± 0.90 | 3.68 ± 0.90 | 3.67 ± 0.91 | 3.67 ± 0.92 | 3.67 ± 0.91 | 3.67 ± 0.92 | 3.66 ± 0.92 | 3.67 ± 0.92 | 3.67 ± 0.92 | 3.67 ± 0.92 |
| Median (IQR) | 3 (3-4) | 3 (3-4) | 3 (3-4) | 3 (3-4) | 3 (3-4) | 3 (3-4) | 3 (3-4) | 3 (3-4) | 3 (3-4) | 3 (3-4) | 3 (3-4) | 3 (3-4) | 3 (3-4) | 3 (3-4) |
| OMID (AMI) | 573 (8.0%) | 578 (8.0%) | 577 (7.9%) | 586 (7.9%) | 589 (7.9%) | 596 (8.0%) | 588 (7.9%) | 587 (7.9%) | 577 (7.8%) | 580 (7.9%) | 582 (7.9%) | 589 (8.0%) | 596 (8.1%) | 593 (8.1%) |
| Enrollment status |  |  |  |  |  |  |  |  |  |  |  |  |  |  |
| Enrolled | 6,764 (94.9%) | 6,878 (94.9%) | 6,958 (94.9%) | 7,054 (94.9%) | 7,072 (94.9%) | 7,085 (95.0%) | 7,046 (94.9%) | 7,057 (95.1%) | 7,055 (95.2%) | 7,035 (95.3%) | 6,952 (94.4%) | 7,003 (94.9%) | 7,006 (95.0%) | 7,000 (95.1%) |
| Virtually enrolled | 364 (5.1%) | 371 (5.1%) | 372 (5.1%) | 377 (5.1%) | 377 (5.1%) | 371 (5.0%) | 378 (5.1%) | 361 (4.9%) | 355 (4.8%) | 350 (4.7%) | 411 (5.6%) | 375 (5.1%) | 371 (5.0%) | 359 (4.9%) |
|  |  |  |  |  |  |  |  |  |  |  |  |  |  |  |
|  |  |  |  |  |  |  |  |  |  |  |  |  |  |  |
|  | **2013Q3** | **2013Q4** | **2014Q1** | **2014Q2** | **2014Q3** | **2014Q4** | **2015Q1** | **2015Q2** | **2015Q3** | **2015Q4** | **2016Q1** | **2016Q2** | **2016Q3** | **2016Q4** |
| CVD | 5,191 (72.8%) | 5,317 (73.3%) | 5,389 (73.5%) | 5,455 (73.4%) | 5,465 (73.4%) | 5,476 (73.4%) | 5,449 (73.4%) | 5,447 (73.4%) | 5,434 (73.3%) | 5,407 (73.2%) | 5,386 (73.1%) | 5,379 (72.9%) | 5,365 (72.7%) | 5,347 (72.7%) |
| CHF | 1,024 (14.4%) | 1,048 (14.5%) | 1,054 (14.4%) | 1,084 (14.6%) | 1,094 (14.7%) | 1,091 (14.6%) | 1,091 (14.7%) | 1,102 (14.9%) | 1,092 (14.7%) | 1,071 (14.5%) | 1,069 (14.5%) | 1,073 (14.5%) | 1,077 (14.6%) | 1,078 (14.6%) |
| OMID (AMI) | 573 (8.0%) | 578 (8.0%) | 577 (7.9%) | 586 (7.9%) | 589 (7.9%) | 596 (8.0%) | 588 (7.9%) | 587 (7.9%) | 577 (7.8%) | 580 (7.9%) | 582 (7.9%) | 589 (8.0%) | 596 (8.1%) | 593 (8.1%) |
| HTN | 5,875 (82.4%) | 5,975 (82.4%) | 6,039 (82.4%) | 6,111 (82.2%) | 6,141 (82.4%) | 6,146 (82.4%) | 6,124 (82.5%) | 6,123 (82.5%) | 6,123 (82.6%) | 6,112 (82.8%) | 6,089 (82.7%) | 6,100 (82.7%) | 6,095 (82.6%) | 6,083 (82.7%) |

ODD: Ontario Diabetes Database; SD: standard deviation; IQR: interquartile range; ADG: adjusted diagnostic groups; RUB: resource utilization band; OMID: Ontario myocardial infarct dataset; AMI: acute myocardial infarct; CVD: cardiovascular disease; CHF: congestive heart failure; HTN: hypertension

*patient had no encounters in the CPCSSN dataset

† RUBs estimate healthcare resource use grouped by morbidity levels: 0=non user to 5 = very high morbidity
