# Supplementary material for: Impact of the diabetes Canada guideline dissemination strategy on dispensed vascular protective medications for older patients in Ontario, Canada: a linked EMR and administrative data study
Source: BMC Health Serv Res. 2020 May 1;20:370. doi: 10.1186/s12913-020-05232-3 (PMC7195730; doi:10.1186/s12913-020-05232-3)
Supplement: Supplementary file 2 — Additional file 2. Supplementary file 2: Rates of medication coverage for patients with diabetes, for all quarters of each year, EMR cohort. [file 12913_2020_5232_MOESM2_ESM.docx]

Supplementary file 2

Rates of medication coverage for patients with diabetes, for all quarters of each year, EMR cohort

| Rate of drug dispensed by quarter, all cohort | | | |  |  |  |  |  |  |  |  |  |  |  |
| --- | --- | --- | --- | --- | --- | --- | --- | --- | --- | --- | --- | --- | --- | --- |
| **Drug** | **2010Q1** | **2010Q2** | **2010Q3** | **2010Q4** | **2011Q1** | **2011Q2** | **2011Q3** | **2011Q4** | **2012Q1** | **2012Q2** | **2012Q3** | **2012Q4** | **2013Q1** | **2013Q2** |
| STATIN | 62.87% | 62.25% | 63.82% | 64.78% | 64.71% | 63.89% | 64.85% | 64.95% | 64.26% | 62.45% | 64.07% | 64.59% | 64.82% | 64.03% |
| ACE INHIBITORS | 43.24% | 42.34% | 43.19% | 43.41% | 43.02% | 42.43% | 42.08% | 41.67% | 41.33% | 39.64% | 40.20% | 40.03% | 39.86% | 39.73% |
| ARB INHIBITORS | 21.07% | 20.83% | 21.37% | 21.41% | 21.43% | 21.88% | 21.98% | 22.38% | 22.25% | 21.87% | 22.34% | 22.24% | 22.23% | 22.07% |
| ACE or ARB | 63.40% | 62.31% | 63.76% | 63.99% | 63.93% | 63.71% | 63.70% | 63.54% | 63.10% | 61.04% | 62.27% | 62.00% | 61.80% | 61.48% |
| ANTIPLATELETS* | 9.39% | 9.15% | 9.04% | 9.08% | 8.84% | 8.90% | 9.09% | 8.81% | 8.48% | 8.24% | 8.42% | 8.09% | 7.83% | 7.81% |
| PPI | 21.85% | 22.08% | 22.95% | 22.87% | 23.25% | 23.57% | 23.70% | 24.36% | 23.79% | 23.17% | 24.09% | 24.34% | 24.83% | 25.06% |
|  |  |  |  |  |  |  |  |  |  |  |  |  |  |  |
| Rate of drug dispensed by quarter, only patients with encounter date in CPCSSN | | | | | |  |  |  |  |  |  |  |  |  |
| **Drug** | **2010Q1** | **2010Q2** | **2010Q3** | **2010Q4** | **2011Q1** | **2011Q2** | **2011Q3** | **2011Q4** | **2012Q1** | **2012Q2** | **2012Q3** | **2012Q4** | **2013Q1** | **2013Q2** |
| STATIN | 63.31% | 62.47% | 63.98% | 64.77% | 64.68% | 63.85% | 64.71% | 64.72% | 64.30% | 62.52% | 64.15% | 64.59% | 64.74% | 63.98% |
| ACE INHIBITORS | 44.24% | 43.31% | 44.28% | 44.29% | 44.06% | 43.52% | 43.15% | 42.64% | 42.32% | 40.67% | 41.30% | 40.94% | 40.85% | 40.50% |
| ARB INHIBITORS | 21.11% | 20.76% | 21.21% | 21.14% | 21.23% | 21.66% | 21.68% | 22.00% | 21.76% | 21.50% | 21.82% | 21.75% | 21.65% | 21.51% |
| ACE or ARB | 64.37% | 63.16% | 64.64% | 64.58% | 64.70% | 64.52% | 64.44% | 64.13% | 63.58% | 61.64% | 62.78% | 62.43% | 62.16% | 61.68% |
| ANTIPLATELETS* | 9.28% | 9.11% | 8.89% | 8.90% | 8.59% | 8.70% | 9.02% | 8.68% | 8.37% | 8.18% | 8.39% | 8.10% | 7.88% | 7.85% |
| PPI | 21.35% | 21.63% | 22.45% | 22.34% | 22.72% | 23.14% | 23.29% | 24.02% | 23.47% | 23.07% | 24.00% | 24.22% | 24.68% | 24.90% |
| *patients with a history of myocardial infarct were excluded  ACEi: angiotensin-converting enzyme inhibitor; ARB: angiotensin receptor blockers; PPI: proton pump inhibitor | | | | | | | | |  |  |  |  |  |  |

| Rate of drug dispensed by quarter, all cohort | | | | |  |  |  |  |  |  |  |  |  |  |
| --- | --- | --- | --- | --- | --- | --- | --- | --- | --- | --- | --- | --- | --- | --- |
| **Drug** | **2013Q3** | **2013Q4** | **2014Q1** | **2014Q2** | **2014Q3** | **2014Q4** | **2015Q1** | **2015Q2** | **2015Q3** | **2015Q4** | **2016Q1** | **2016Q2** | **2016Q3** | **2016Q4** |
| STATIN | 64.17% | 64.48% | 63.97% | 63.64% | 64.24% | 64.06% | 64.01% | 63.67% | 64.12% | 64.52% | 65.12% | 64.45% | 64.65% | 65.33% |
| ACE INHIBITORS | 39.48% | 39.37% | 38.72% | 38.39% | 38.34% | 38.30% | 37.97% | 37.88% | 38.07% | 37.94% | 37.48% | 37.11% | 37.26% | 37.21% |
| ARB INHIBITORS | 22.25% | 22.35% | 22.62% | 22.37% | 22.47% | 22.41% | 22.43% | 22.46% | 22.59% | 22.69% | 22.87% | 22.85% | 22.60% | 22.68% |
| ACE or ARB | 61.46% | 61.50% | 61.20% | 60.57% | 60.68% | 60.61% | 60.34% | 60.23% | 60.53% | 60.53% | 60.36% | 59.99% | 59.92% | 59.93% |
| ANTIPLATELETS* | 7.67% | 7.63% | 7.39% | 6.84% | 6.90% | 6.85% | 6.58% | 6.68% | 6.85% | 6.79% | 6.49% | 6.41% | 5.99% | 6.07% |
| PPI | 25.27% | 25.59% | 25.36% | 25.68% | 26.22% | 26.78% | 27.32% | 26.77% | 27.33% | 27.76% | 27.65% | 27.12% | 26.94% | 26.92% |
|  |  |  |  |  |  |  |  |  |  |  |  |  |  |  |
| Rate of drug dispensed by quarter, only patients with encounter date in CPCSSN | | | | | | |  |  |  |  |  |  |  |  |
| **Drug** | **2013Q3** | **2013Q4** | **2014Q1** | **2014Q2** | **2014Q3** | **2014Q4** | **2015Q1** | **2015Q2** | **2015Q3** | **2015Q4** | **2016Q1** | **2016Q2** | **2016Q3** | **2016Q4** |
| STATIN | 64.08% | 64.20% | 63.69% | 63.57% | 64.00% | 63.79% | 63.74% | 63.40% | 63.73% | 64.18% | 64.81% | 64.14% | 64.32% | 64.83% |
| ACE INHIBITORS | 40.24% | 40.11% | 39.61% | 39.14% | 39.18% | 39.18% | 38.85% | 38.68% | 38.79% | 38.67% | 38.26% | 37.90% | 38.08% | 37.91% |
| ARB INHIBITORS | 21.66% | 21.75% | 21.99% | 21.71% | 21.75% | 21.73% | 21.73% | 21.65% | 21.86% | 21.89% | 22.09% | 21.98% | 21.94% | 21.93% |
| ACE or ARB | 61.61% | 61.63% | 61.45% | 60.68% | 60.77% | 60.76% | 60.49% | 60.21% | 60.49% | 60.46% | 60.33% | 59.92% | 60.06% | 59.91% |
| ANTIPLATELETS* | 7.71% | 7.67% | 7.44% | 6.89% | 6.93% | 6.91% | 6.66% | 6.68% | 6.90% | 6.83% | 6.56% | 6.49% | 6.10% | 6.15% |
| PPI | 25.13% | 25.58% | 25.46% | 25.78% | 26.29% | 26.85% | 27.39% | 26.79% | 27.17% | 27.70% | 27.68% | 27.26% | 27.17% | 27.10% |
| *patients with a history of myocardial infarct were excluded  ACEi: angiotensin-converting enzyme inhibitor; ARB: angiotensin receptor blockers; PPI: proton pump inhibitor | | | | | | | | | |  |  |  |  |  |
