# Supplementary material for: Impact of the diabetes Canada guideline dissemination strategy on dispensed vascular protective medications for older patients in Ontario, Canada: a linked EMR and administrative data study
Source: BMC Health Serv Res. 2020 May 1;20:370. doi: 10.1186/s12913-020-05232-3 (PMC7195730; doi:10.1186/s12913-020-05232-3)
Supplement: Supplementary file 3 — Additional file 3. Supplementary file 3. Segmented regression analysis for vascular protective medication and comparator coverage, 2010 to 2016, EMR cohort, for patients with at least one encounter. [file 12913_2020_5232_MOESM3_ESM.docx]

Supplementary file 3

Segmented regression analysis for vascular protective medication and comparator coverage, 2010 to 2016, EMR cohort, for patients with at least one encounter

| **Variable** | **Estimate** | **SE** | **Pvalue** |
| --- | --- | --- | --- |
| Statin |  |  |  |
| Intercept | 63.52 | 0.44 | <.0001 |
| Pre-intervention trend | 0.08 | 0.05 | 0.18 |
| guideline | -0.86 | 0.54 | 0.13 |
| Trend change | -0.03 | 0.07 | 0.70 |
| ACEi or ARB |  |  |  |
| Intercept | 64.97 | 0.42 | <.0001 |
| Pre-intervention trend | -0.20 | 0.05 | 0.00 |
| guideline | -0.64 | 0.50 | 0.21 |
| Trend change | 0.06 | 0.07 | 0.38 |
| ANTIPLATELETS |  |  |  |
| Intercept | 9.35 | 0.14 | <.0001 |
| Pre-intervention trend | -0.11 | 0.02 | <.0001 |
| guideline | -0.12 | 0.17 | 0.48 |
| Trend change | -0.01 | 0.02 | 0.67 |
| PPI |  |  |  |
| Intercept | 21.27 | 0.38 | <.0001 |
| Pre-intervention trend | 0.27 | 0.04 | <.0001 |
| guideline | 0.30 | 0.39 | 0.46 |
| Trend change | -0.08 | 0.06 | 0.20 |

SE: standard error

ACEi: angiotensin-converting enzyme inhibitor; ARB: angiotensin receptor blockers; PPI: proton pump inhibitor
