# Supplementary material for: Impact of the diabetes Canada guideline dissemination strategy on dispensed vascular protective medications for older patients in Ontario, Canada: a linked EMR and administrative data study
Source: BMC Health Serv Res. 2020 May 1;20:370. doi: 10.1186/s12913-020-05232-3 (PMC7195730; doi:10.1186/s12913-020-05232-3)
Supplement: Supplementary file 4 — Additional file 4. Supplementary file 4. Patient characteristics for all quarters of interest, administrative cohort. [file 12913_2020_5232_MOESM4_ESM.docx]

Supplementary file 4

Patient characteristics for all quarters of interest, administrative cohort

|  | **2010Q1** | **2010Q2** | **2010Q3** | **2010Q4** | **2011Q1** | **2011Q2** | **2011Q3** | **2011Q4** | **2012Q1** | **2012Q2** | **2012Q3** | **2012Q4** | **2013Q1** | **2013Q2** |
| --- | --- | --- | --- | --- | --- | --- | --- | --- | --- | --- | --- | --- | --- | --- |
| TOTAL | N=443,608 | N=449,096 | N=456,467 | N=461,203 | N=466,092 | N=471,858 | N=478,367 | N=483,064 | N=488,187 | N=494,112 | N=501,646 | N=507,644 | N=513,737 | N=521,020 |
| Age |  |  |  |  |  |  |  |  |  |  |  |  |  |  |
| Mean ± SD | 75.60 ± 6.84 | 75.61 ± 6.86 | 75.63 ± 6.88 | 75.64 ± 6.89 | 75.65 ± 6.91 | 75.68 ± 6.93 | 75.70 ± 6.94 | 75.72 ± 6.96 | 75.73 ± 6.97 | 75.75 ± 6.99 | 75.75 ± 7.02 | 75.73 ± 7.04 | 75.70 ± 7.06 | 75.70 ± 7.09 |
| Median (IQR) | 75 (70-80) | 75 (70-80) | 75 (70-80) | 75 (70-80) | 75 (70-80) | 75 (70-80) | 75 (70-81) | 75 (70-81) | 75 (70-81) | 75 (70-81) | 75 (70-81) | 75 (70-81) | 75 (70-81) | 75 (70-81) |
| Sex |  |  |  |  |  |  |  |  |  |  |  |  |  |  |
| Female | 223,874 (50.5%) | 226,364 (50.4%) | 229,774 (50.3%) | 231,914 (50.3%) | 234,173 (50.2%) | 236,842 (50.2%) | 239,943 (50.2%) | 242,216 (50.1%) | 244,748 (50.1%) | 247,493 (50.1%) | 250,972 (50.0%) | 253,844 (50.0%) | 256,606 (49.9%) | 259,986 (49.9%) |
| Male | 219,734 (49.5%) | 222,732 (49.6%) | 226,693 (49.7%) | 229,289 (49.7%) | 231,919 (49.8%) | 235,016 (49.8%) | 238,424 (49.8%) | 240,848 (49.9%) | 243,439 (49.9%) | 246,619 (49.9%) | 250,674 (50.0%) | 253,800 (50.0%) | 257,131 (50.1%) | 261,034 (50.1%) |
| Income quintile |  |  |  |  |  |  |  |  |  |  |  |  |  |  |
| Q1 (lowest) | 95,488 (21.5%) | 96,194 (21.4%) | 97,330 (21.3%) | 98,033 (21.3%) | 98,510 (21.1%) | 99,448 (21.1%) | 100,577 (21.0%) | 101,272 (21.0%) | 102,097 (20.9%) | 102,956 (20.8%) | 104,260 (20.8%) | 105,203 (20.7%) | 106,099 (20.7%) | 107,267 (20.6%) |
| Q2 | 97,017 (21.9%) | 98,151 (21.9%) | 99,747 (21.9%) | 100,687 (21.8%) | 101,488 (21.8%) | 102,602 (21.7%) | 104,003 (21.7%) | 105,034 (21.7%) | 105,691 (21.6%) | 107,034 (21.7%) | 108,636 (21.7%) | 109,882 (21.6%) | 110,832 (21.6%) | 112,360 (21.6%) |
| Q3 | 88,583 (20.0%) | 89,792 (20.0%) | 91,290 (20.0%) | 92,325 (20.0%) | 93,564 (20.1%) | 94,780 (20.1%) | 96,101 (20.1%) | 97,083 (20.1%) | 98,297 (20.1%) | 99,453 (20.1%) | 101,062 (20.1%) | 102,265 (20.1%) | 103,392 (20.1%) | 104,865 (20.1%) |
| Q4 | 85,599 (19.3%) | 86,845 (19.3%) | 88,398 (19.4%) | 89,459 (19.4%) | 90,808 (19.5%) | 92,129 (19.5%) | 93,517 (19.5%) | 94,536 (19.6%) | 95,852 (19.6%) | 97,152 (19.7%) | 98,819 (19.7%) | 100,120 (19.7%) | 101,912 (19.8%) | 103,573 (19.9%) |
| Q5 (highest) | 75,179 (16.9%) | 76,360 (17.0%) | 77,941 (17.1%) | 78,944 (17.1%) | 79,898 (17.1%) | 81,070 (17.2%) | 82,344 (17.2%) | 83,307 (17.2%) | 84,350 (17.3%) | 85,623 (17.3%) | 86,958 (17.3%) | 88,263 (17.4%) | 89,468 (17.4%) | 90,907 (17.4%) |
| Missing | 1,742 (0.4%) | 1,754 (0.4%) | 1,761 (0.4%) | 1,755 (0.4%) | 1,824 (0.4%) | 1,829 (0.4%) | 1,825 (0.4%) | 1,832 (0.4%) | 1,900 (0.4%) | 1,894 (0.4%) | 1,911 (0.4%) | 1,911 (0.4%) | 2,034 (0.4%) | 2,048 (0.4%) |
| Rural | 65,408 (14.7%) | 65,971 (14.7%) | 67,347 (14.8%) | 68,225 (14.8%) | 68,458 (14.7%) | 69,441 (14.7%) | 70,647 (14.8%) | 71,484 (14.8%) | 71,660 (14.7%) | 72,568 (14.7%) | 73,854 (14.7%) | 74,920 (14.8%) | 75,092 (14.6%) | 76,291 (14.6%) |
| ADG |  |  |  |  |  |  |  |  |  |  |  |  |  |  |
| Mean ± SD | 7.90 ± 3.83 | 7.93 ± 3.83 | 7.91 ± 3.83 | 7.91 ± 3.83 | 7.89 ± 3.83 | 7.90 ± 3.84 | 7.90 ± 3.84 | 7.91 ± 3.85 | 7.90 ± 3.86 | 7.91 ± 3.87 | 7.89 ± 3.87 | 7.90 ± 3.88 | 7.89 ± 3.88 | 7.88 ± 3.89 |
| Median (IQR) | 8 (5-10) | 8 (5-10) | 8 (5-10) | 8 (5-10) | 8 (5-10) | 8 (5-10) | 8 (5-10) | 8 (5-10) | 8 (5-10) | 8 (5-10) | 8 (5-10) | 8 (5-10) | 8 (5-10) | 8 (5-10) |
| RUB* |  |  |  |  |  |  |  |  |  |  |  |  |  |  |
| Mean ± SD | 3.65 ± 0.90 | 3.66 ± 0.89 | 3.65 ± 0.90 | 3.65 ± 0.90 | 3.65 ± 0.90 | 3.65 ± 0.90 | 3.65 ± 0.90 | 3.65 ± 0.90 | 3.65 ± 0.90 | 3.65 ± 0.90 | 3.65 ± 0.90 | 3.65 ± 0.90 | 3.64 ± 0.90 | 3.64 ± 0.91 |
| Median (IQR) | 3 (3-4) | 3 (3-4) | 3 (3-4) | 3 (3-4) | 3 (3-4) | 3 (3-4) | 3 (3-4) | 3 (3-4) | 3 (3-4) | 3 (3-4) | 3 (3-4) | 3 (3-4) | 3 (3-4) | 3 (3-4) |
| OMID (AMI) | 37,332 (8.4%) | 37,802 (8.4%) | 38,371 (8.4%) | 38,684 (8.4%) | 39,015 (8.4%) | 39,495 (8.4%) | 40,071 (8.4%) | 40,437 (8.4%) | 40,729 (8.3%) | 41,217 (8.3%) | 41,776 (8.3%) | 42,229 (8.3%) | 42,550 (8.3%) | 43,109 (8.3%) |
| Enrollment status |  |  |  |  |  |  |  |  |  |  |  |  |  |  |
| Enrolled | 374,436 (84.4%) | 379,747 (84.6%) | 387,841 (85.0%) | 393,950 (85.4%) | 399,380 (85.7%) | 405,698 (86.0%) | 412,535 (86.2%) | 418,699 (86.7%) | 424,893 (87.0%) | 431,643 (87.4%) | 439,225 (87.6%) | 444,911 (87.6%) | 451,284 (87.8%) | 458,970 (88.1%) |
| Virtually enrolled | 69,172 (15.6%) | 69,349 (15.4%) | 68,626 (15.0%) | 67,253 (14.6%) | 66,712 (14.3%) | 66,160 (14.0%) | 65,832 (13.8%) | 64,365 (13.3%) | 63,294 (13.0%) | 62,469 (12.6%) | 62,421 (12.4%) | 62,733 (12.4%) | 62,453 (12.2%) | 62,050 (11.9%) |
|  |  |  |  |  |  |  |  |  |  |  |  |  |  |  |
|  |  |  |  |  |  |  |  |  |  |  |  |  |  |  |
|  |  |  |  |  |  |  |  |  |  |  |  |  |  |  |
|  | **2010Q1** | **2010Q2** | **2010Q3** | **2010Q4** | **2011Q1** | **2011Q2** | **2011Q3** | **2011Q4** | **2012Q1** | **2012Q2** | **2012Q3** | **2012Q4** | **2013Q1** | **2013Q2** |
| Cardiovascular condition | 381,896 (86.1%) | 387,072 (86.2%) | 393,629 (86.2%) | 398,119 (86.3%) | 402,524 (86.4%) | 407,842 (86.4%) | 413,655 (86.5%) | 418,009 (86.5%) | 422,519 (86.5%) | 427,803 (86.6%) | 434,291 (86.6%) | 439,590 (86.6%) | 444,766 (86.6%) | 451,112 (86.6%) |
| CHF | 70,343 (15.9%) | 70,953 (15.8%) | 71,793 (15.7%) | 72,242 (15.7%) | 72,678 (15.6%) | 73,593 (15.6%) | 74,376 (15.5%) | 74,900 (15.5%) | 75,397 (15.4%) | 76,240 (15.4%) | 76,973 (15.3%) | 77,552 (15.3%) | 78,039 (15.2%) | 78,969 (15.2%) |
| OMID (AMI) | 37,332 (8.4%) | 37,802 (8.4%) | 38,371 (8.4%) | 38,684 (8.4%) | 39,015 (8.4%) | 39,495 (8.4%) | 40,071 (8.4%) | 40,437 (8.4%) | 40,729 (8.3%) | 41,217 (8.3%) | 41,776 (8.3%) | 42,229 (8.3%) | 42,550 (8.3%) | 43,109 (8.3%) |
| HTN | 374,702 (84.5%) | 379,942 (84.6%) | 386,480 (84.7%) | 390,990 (84.8%) | 395,440 (84.8%) | 400,800 (84.9%) | 406,641 (85.0%) | 411,080 (85.1%) | 415,646 (85.1%) | 420,952 (85.2%) | 427,464 (85.2%) | 432,814 (85.3%) | 438,015 (85.3%) | 444,369 (85.3%) |

ODD: Ontario Diabetes Database; SD: standard deviation; IQR: interquartile range; ADG: adjusted diagnostic groups; RUB: resource utilization band; OMID: Ontario myocardial infarct dataset; AMI: acute myocardial infarct; CHR: congestive heart failure; HTN: hypertension

* RUBs estimate healthcare resource use grouped by morbidity levels: 0=non user to 5 = very high morbidity

|  | **2013Q3** | **2013Q4** | **2014Q1** | **2014Q2** | **2014Q3** | **2014Q4** | **2015Q1** | **2015Q2** | **2015Q3** | **2015Q4** | **2016Q1** | **2016Q2** | **2016Q3** | **2016Q4** |
| --- | --- | --- | --- | --- | --- | --- | --- | --- | --- | --- | --- | --- | --- | --- |
| TOTAL | N=529,842 | N=537,132 | N=544,224 | N=551,517 | N=559,670 | N=565,805 | N=571,800 | N=578,557 | N=586,450 | N=592,921 | N=599,122 | N=606,198 | N=614,256 | N=619,902 |
| Age |  |  |  |  |  |  |  |  |  |  |  |  |  |  |
| Mean ± SD | 75.69 ± 7.12 | 75.67 ± 7.14 | 75.66 ± 7.15 | 75.66 ± 7.17 | 75.67 ± 7.18 | 75.66 ± 7.19 | 75.65 ± 7.19 | 75.66 ± 7.21 | 75.67 ± 7.22 | 75.67 ± 7.23 | 75.67 ± 7.23 | 75.68 ± 7.25 | 75.70 ± 7.25 | 75.70 ± 7.25 |
| Median (IQR) | 75 (70-81) | 75 (70-81) | 75 (70-81) | 75 (70-81) | 75 (70-81) | 75 (70-81) | 75 (70-81) | 74 (70-81) | 74 (70-81) | 74 (69-81) | 74 (69-81) | 74 (69-81) | 74 (70-81) | 74 (70-81) |
| Sex |  |  |  |  |  |  |  |  |  |  |  |  |  |  |
| Female | 264,138 (49.9%) | 267,552 (49.8%) | 270,995 (49.8%) | 274,413 (49.8%) | 278,360 (49.7%) | 281,314 (49.7%) | 284,190 (49.7%) | 287,254 (49.7%) | 291,030 (49.6%) | 294,218 (49.6%) | 297,109 (49.6%) | 300,501 (49.6%) | 304,293 (49.5%) | 307,003 (49.5%) |
| Male | 265,704 (50.1%) | 269,580 (50.2%) | 273,229 (50.2%) | 277,104 (50.2%) | 281,310 (50.3%) | 284,491 (50.3%) | 287,610 (50.3%) | 291,303 (50.3%) | 295,420 (50.4%) | 298,703 (50.4%) | 302,013 (50.4%) | 305,697 (50.4%) | 309,963 (50.5%) | 312,899 (50.5%) |
| Income quintile |  |  |  |  |  |  |  |  |  |  |  |  |  |  |
| Q1 (lowest) | 108,786 (20.5%) | 109,849 (20.5%) | 111,256 (20.4%) | 112,505 (20.4%) | 113,805 (20.3%) | 114,708 (20.3%) | 115,641 (20.2%) | 116,723 (20.2%) | 118,084 (20.1%) | 118,953 (20.1%) | 120,280 (20.1%) | 121,496 (20.0%) | 122,829 (20.0%) | 123,717 (20.0%) |
| Q2 | 114,275 (21.6%) | 115,825 (21.6%) | 116,671 (21.4%) | 118,171 (21.4%) | 119,979 (21.4%) | 121,192 (21.4%) | 121,898 (21.3%) | 123,336 (21.3%) | 124,946 (21.3%) | 126,315 (21.3%) | 127,241 (21.2%) | 128,622 (21.2%) | 130,258 (21.2%) | 131,286 (21.2%) |
| Q3 | 106,683 (20.1%) | 108,249 (20.2%) | 109,813 (20.2%) | 111,274 (20.2%) | 112,999 (20.2%) | 114,359 (20.2%) | 115,603 (20.2%) | 117,130 (20.2%) | 118,755 (20.2%) | 120,095 (20.3%) | 121,180 (20.2%) | 122,681 (20.2%) | 124,407 (20.3%) | 125,609 (20.3%) |
| Q4 | 105,378 (19.9%) | 106,951 (19.9%) | 108,692 (20.0%) | 110,261 (20.0%) | 112,085 (20.0%) | 113,587 (20.1%) | 115,226 (20.2%) | 116,651 (20.2%) | 118,413 (20.2%) | 119,969 (20.2%) | 121,499 (20.3%) | 123,128 (20.3%) | 124,993 (20.3%) | 126,250 (20.4%) |
| Q5 (highest) | 92,675 (17.5%) | 94,204 (17.5%) | 95,666 (17.6%) | 97,180 (17.6%) | 98,668 (17.6%) | 99,822 (17.6%) | 101,179 (17.7%) | 102,450 (17.7%) | 103,982 (17.7%) | 105,318 (17.8%) | 106,523 (17.8%) | 107,860 (17.8%) | 109,348 (17.8%) | 110,619 (17.8%) |
| Missing | 2,045 (0.4%) | 2,054 (0.4%) | 2,126 (0.4%) | 2,126 (0.4%) | 2,134 (0.4%) | 2,137 (0.4%) | 2,253 (0.4%) | 2,267 (0.4%) | 2,270 (0.4%) | 2,271 (0.4%) | 2,399 (0.4%) | 2,411 (0.4%) | 2,421 (0.4%) | 2,421 (0.4%) |
| Rural | 77,647 (14.7%) | 78,719 (14.7%) | 79,026 (14.5%) | 80,068 (14.5%) | 81,352 (14.5%) | 82,296 (14.5%) | 82,247 (14.4%) | 83,165 (14.4%) | 84,368 (14.4%) | 85,464 (14.4%) | 85,540 (14.3%) | 86,587 (14.3%) | 87,788 (14.3%) | 88,621 (14.3%) |
| ADG |  |  |  |  |  |  |  |  |  |  |  |  |  |  |
| Mean ± SD | 7.88 ± 3.90 | 7.88 ± 3.91 | 7.84 ± 3.92 | 7.81 ± 3.93 | 7.82 ± 3.94 | 7.83 ± 3.95 | 7.78 ± 3.95 | 7.80 ± 3.96 | 7.80 ± 3.96 | 7.82 ± 3.97 | 7.81 ± 3.97 | 7.83 ± 3.98 | 7.84 ± 3.98 | 7.84 ± 3.98 |
| Median (IQR) | 8 (5-10) | 8 (5-10) | 7 (5-10) | 7 (5-10) | 7 (5-10) | 7 (5-10) | 7 (5-10) | 7 (5-10) | 7 (5-10) | 7 (5-10) | 7 (5-10) | 7 (5-10) | 7 (5-10) | 7 (5-10) |
| RUB |  |  |  |  |  |  |  |  |  |  |  |  |  |  |
| Mean ± SD | 3.64 ± 0.91 | 3.64 ± 0.91 | 3.64 ± 0.91 | 3.64 ± 0.91 | 3.64 ± 0.91 | 3.65 ± 0.91 | 3.64 ± 0.91 | 3.64 ± 0.91 | 3.65 ± 0.91 | 3.65 ± 0.91 | 3.65 ± 0.92 | 3.65 ± 0.92 | 3.65 ± 0.92 | 3.65 ± 0.92 |
| Median (IQR) | 3 (3-4) | 3 (3-4) | 3 (3-4) | 3 (3-4) | 3 (3-4) | 3 (3-4) | 3 (3-4) | 3 (3-4) | 3 (3-4) | 3 (3-4) | 3 (3-4) | 3 (3-4) | 3 (3-4) | 3 (3-4) |
| OMID (AMI) | 43,710 (8.2%) | 44,266 (8.2%) | 44,579 (8.2%) | 45,166 (8.2%) | 45,776 (8.2%) | 46,208 (8.2%) | 46,463 (8.1%) | 46,992 (8.1%) | 47,572 (8.1%) | 48,048 (8.1%) | 48,452 (8.1%) | 49,048 (8.1%) | 49,661 (8.1%) | 50,053 (8.1%) |
| Enrollment status |  |  |  |  |  |  |  |  |  |  |  |  |  |  |
| Enrolled | 467,734 (88.3%) | 475,388 (88.5%) | 482,316 (88.6%) | 489,742 (88.8%) | 497,196 (88.8%) | 504,203 (89.1%) | 510,631 (89.3%) | 518,630 (89.6%) | 526,194 (89.7%) | 532,759 (89.9%) | 538,094 (89.8%) | 543,955 (89.7%) | 551,522 (89.8%) | 555,273 (89.6%) |
| Virtually enrolled | 62,108 (11.7%) | 61,744 (11.5%) | 61,908 (11.4%) | 61,775 (11.2%) | 62,474 (11.2%) | 61,602 (10.9%) | 61,169 (10.7%) | 59,927 (10.4%) | 60,256 (10.3%) | 60,162 (10.1%) | 61,028 (10.2%) | 62,243 (10.3%) | 62,734 (10.2%) | 64,629 (10.4%) |
|  |  |  |  |  |  |  |  |  |  |  |  |  |  |  |
|  |  |  |  |  |  |  |  |  |  |  |  |  |  |  |
|  |  |  |  |  |  |  |  |  |  |  |  |  |  |  |
|  | **2013Q3** | **2013Q4** | **2014Q1** | **2014Q2** | **2014Q3** | **2014Q4** | **2015Q1** | **2015Q2** | **2015Q3** | **2015Q4** | **2016Q1** | **2016Q2** | **2016Q3** | **2016Q4** |
| Cardiovascular condition | 458,447 (86.5%) | 464,653 (86.5%) | 470,445 (86.4%) | 476,642 (86.4%) | 483,446 (86.4%) | 488,525 (86.3%) | 493,342 (86.3%) | 498,962 (86.2%) | 505,459 (86.2%) | 510,804 (86.2%) | 515,653 (86.1%) | 521,329 (86.0%) | 527,717 (85.9%) | 531,989 (85.8%) |
| CHF | 80,018 (15.1%) | 80,745 (15.0%) | 81,389 (15.0%) | 82,560 (15.0%) | 83,586 (14.9%) | 84,266 (14.9%) | 84,822 (14.8%) | 85,891 (14.8%) | 86,820 (14.8%) | 87,444 (14.7%) | 87,876 (14.7%) | 88,837 (14.7%) | 89,534 (14.6%) | 89,868 (14.5%) |
| OMID (AMI) | 43,710 (8.2%) | 44,266 (8.2%) | 44,579 (8.2%) | 45,166 (8.2%) | 45,776 (8.2%) | 46,208 (8.2%) | 46,463 (8.1%) | 46,992 (8.1%) | 47,572 (8.1%) | 48,048 (8.1%) | 48,452 (8.1%) | 49,048 (8.1%) | 49,661 (8.1%) | 50,053 (8.1%) |
| HTN | 451,639 (85.2%) | 457,873 (85.2%) | 463,672 (85.2%) | 469,871 (85.2%) | 476,663 (85.2%) | 481,728 (85.1%) | 486,454 (85.1%) | 492,022 (85.0%) | 498,453 (85.0%) | 503,740 (85.0%) | 508,602 (84.9%) | 514,235 (84.8%) | 520,531 (84.7%) | 524,725 (84.6%) |

ODD: Ontario Diabetes Database; SD: standard deviation; IQR: interquartile range; ADG: adjusted diagnostic groups; RUB: resource utilization band; OMID: Ontario myocardial infarct dataset; AMI: acute myocardial infarct; CHR: congestive heart failure; HTN: hypertension

* RUBs estimate healthcare resource use grouped by morbidity levels: 0=non user to 5 = very high morbidity
