# Supplementary material for: Impact of the diabetes Canada guideline dissemination strategy on dispensed vascular protective medications for older patients in Ontario, Canada: a linked EMR and administrative data study
Source: BMC Health Serv Res. 2020 May 1;20:370. doi: 10.1186/s12913-020-05232-3 (PMC7195730; doi:10.1186/s12913-020-05232-3)
Supplement: Supplementary file 5 — Additional file 5. Supplementary file 5. Rates of medication coverage for patients with diabetes, for all quarters, 2010 to 2016, administrative cohort. [file 12913_2020_5232_MOESM5_ESM.docx]

Supplementary file 5

Rates of medication coverage for patients with diabetes, for all quarters, 2010 to 2016, administrative cohort

|  | | | |  |  |  |  |  |  |  |  |  |  |  |
| --- | --- | --- | --- | --- | --- | --- | --- | --- | --- | --- | --- | --- | --- | --- |
| **Drug** | **2010Q1** | **2010Q2** | **2010Q3** | **2010Q4** | **2011Q1** | **2011Q2** | **2011Q3** | **2011Q4** | **2012Q1** | **2012Q2** | **2012Q3** | **2012Q4** | **2013Q1** | **2013Q2** |
| STATIN | 60.91% | 60.85% | 61.56% | 62.19% | 62.62% | 62.41% | 62.73% | 63.14% | 62.83% | 61.28% | 63.09% | 63.59% | 63.76% | 63.33% |
| ACE INHIBITORS | 38.00% | 37.44% | 37.15% | 36.82% | 36.48% | 35.99% | 35.72% | 35.48% | 34.95% | 33.88% | 34.38% | 34.21% | 33.96% | 33.60% |
| ARB INHIBITORS | 25.76% | 25.82% | 26.18% | 26.49% | 26.62% | 26.65% | 26.81% | 26.91% | 26.79% | 26.27% | 27.09% | 27.34% | 27.42% | 27.33% |
| ACEi or ARB | 61.94% | 61.62% | 61.80% | 61.87% | 61.80% | 61.44% | 61.42% | 61.39% | 60.87% | 59.44% | 60.71% | 60.82% | 60.72% | 60.34% |
| ANTIPLATELETS | 10.23% | 10.10% | 10.07% | 10.03% | 9.97% | 9.85% | 9.79% | 9.66% | 9.40% | 9.01% | 9.12% | 8.99% | 8.79% | 8.60% |
| PPI | 22.92% | 22.98% | 23.28% | 23.64% | 23.91% | 24.02% | 24.27% | 24.64% | 24.57% | 24.20% | 25.01% | 25.39% | 25.56% | 25.50% |

| **Drug** | **2013Q3** | **2013Q4** | **2014Q1** | **2014Q2** | **2014Q3** | **2014Q4** | **2015Q1** | **2015Q2** | **2015Q3** | **2015Q4** | **2016Q1** | **2016Q2** | **2016Q3** | **2016Q4** |
| --- | --- | --- | --- | --- | --- | --- | --- | --- | --- | --- | --- | --- | --- | --- |
| STATIN | 63.43% | 63.73% | 63.83% | 63.45% | 63.91% | 64.16% | 64.18% | 63.70% | 64.00% | 64.31% | 64.48% | 64.12% | 64.41% | 64.76% |
| ACE INHIBITORS | 33.37% | 33.17% | 32.88% | 32.53% | 32.45% | 32.30% | 32.10% | 31.74% | 31.65% | 31.48% | 31.39% | 31.13% | 31.03% | 30.80% |
| ARB INHIBITORS | 27.46% | 27.60% | 27.86% | 27.76% | 27.96% | 27.98% | 27.89% | 27.61% | 27.69% | 27.77% | 27.95% | 27.87% | 27.94% | 27.93% |
| ACEi or ARB | 60.32% | 60.27% | 60.30% | 59.92% | 60.06% | 59.98% | 59.72% | 59.13% | 59.13% | 59.06% | 59.16% | 58.84% | 58.85% | 58.64% |
| ANTIPLATELETS | 8.49% | 8.35% | 8.14% | 7.90% | 7.81% | 7.65% | 7.48% | 7.30% | 7.16% | 7.02% | 6.91% | 6.75% | 6.63% | 6.49% |
| PPI | 25.74% | 26.02% | 26.14% | 26.08% | 26.38% | 26.59% | 26.72% | 26.64% | 26.92% | 27.21% | 27.34% | 26.98% | 26.94% | 26.95% |

*patients with a history of myocardial infarct were excluded

ACEi: angiotensin-converting enzyme inhibitor; ARB: angiotensin receptor blockers; PPI: proton pump
